# Supplementary material for: Phagocytosis-initiated tumor hybrid cells acquire a c-Myc-mediated quasi-polarization state for immunoevasion and distant dissemination
Source: Nat Commun. 2023 Oct 17;14:6569. doi: 10.1038/s41467-023-42303-5 (PMC10582093; doi:10.1038/s41467-023-42303-5)
Supplement: Supplementary file 1 — Supplementary Information [file 41467_2023_42303_MOESM1_ESM.pdf]

Supplementary information

Phagocytosis-initiated tumor hybrid cells acquire a c-Myc-mediated quasi-polarization state for immunoevasion and distant dissemination

Chih-Wei Chou, Chia-Nung Hung, Cheryl Hsiang-Ling Chiu, Xi Tan, Meizhen Chen, Chien-Chin Chen, Moawiz Saeed, Che-Wei Hsu, Michael A. Liss, Chiou-Miin Wang, Zhao Lai, Nathaniel Alvarez, Pawel Osmulski, Maria E. Gaczynska, Li-Ling Lin, Veronica Ortega, Nameer B. Kirma, Kexin Xu, Zhijie Liu, A. Pratap. Kumar, Josephine A. Taverna, Gopalrao V. N. Velagaleti, Chun-Liang Chen, Zhao Zhang, and Tim Hui-Ming Huang

Supplementary Fig. 1 (Related to Fig 1)

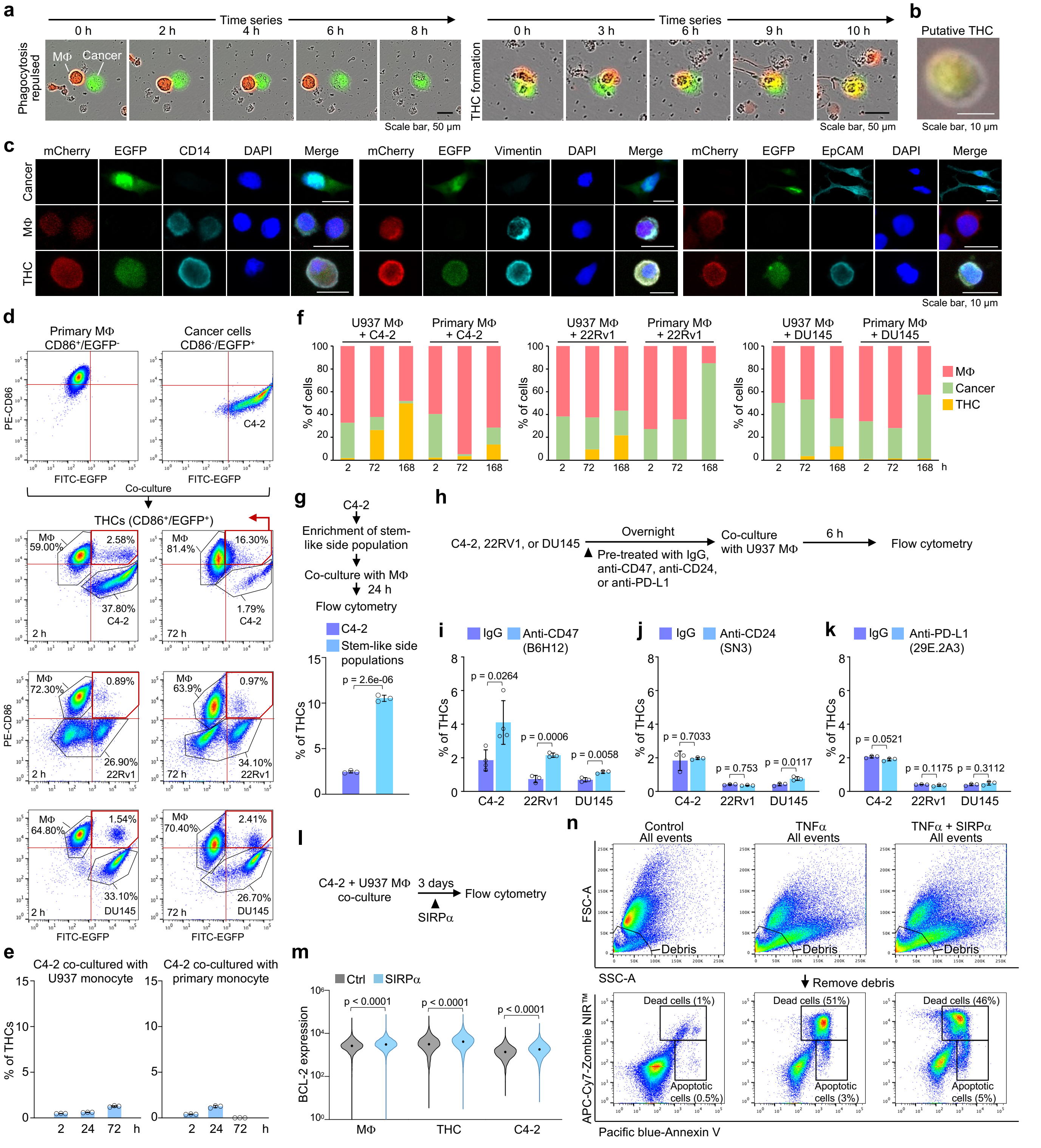

Supplementary Fig. 1. Tumor hybrid cells (THCs) generated through phagocytosis in co-cultured macrophages and cancer cells. Related to Fig. 1

a Time-lapse live images of a U937 macrophage (mCherry) and a C4-2 cancer cell (EGFP) in the co-culture model. *Left*: repulsed phagocytosis. *Right*: THC formation. Scale bar, 50 μm. **b** A THC sorted by fluorescence-activated cell sorting (FACS) co-expressing both mCherry and EGFP. **c** Representative immunofluorescent images of the monocyte/macrophage marker CD14, the mesenchymal marker Vimentin, and the epithelial marker EpCAM in parental C4-2 cells, U937 macrophages, and THCs sorted by FACS. Scale bar, 10 μm. **d** A presentative example using flow cytometry to identify THCs in co-cultures of human primary macrophages and prostate cancer cell lines C4-2, 22Rv1, or DU145. **e** Percentages of THCs in C4-2 cells co-cultured with U937 monocytes (*left*) or human primary monocytes (*right*). **f** The proportions of macrophages (red), cancer cells (green), and THCs (yellow) in different co-culture models at 2, 72, 168 hours. A higher proportion of THCs was shown in C4-2 co-culture models and the number of THCs was accumulated over time. **g** Proportion of THCs in U937 macrophages co-cultured with untreated C4-2 or enriched C4-2 stem-like side population cells for 24 hours. **h** Workflow of cancer cells pretreated with inhibitors of don't-eat-me signals before co-culture. **i-k** Bar graphs showing the effects of anti-CD47 (i), anti-CD24 (j), or anti-PD-L1 (k) on the formation of THCs ($CD86^+$/$EGFP^+$) examined by flow cytometry. **l**, **m** Flow cytometry analysis of the BCL-2 expression in the co-cultured cells with or without SIRPα treatment. **n** Flow cytometry gating strategy for identifying the dead or apoptotic cells in Fig. 1m. Images represent three (a) and two (c) independent experiments. Data are the mean $\pm$ SD of three independent experiments. *P*-values were determined using a two-sided unpaired *t*-test. Source data for e-g, i-k, m are provided as a Source Data file.

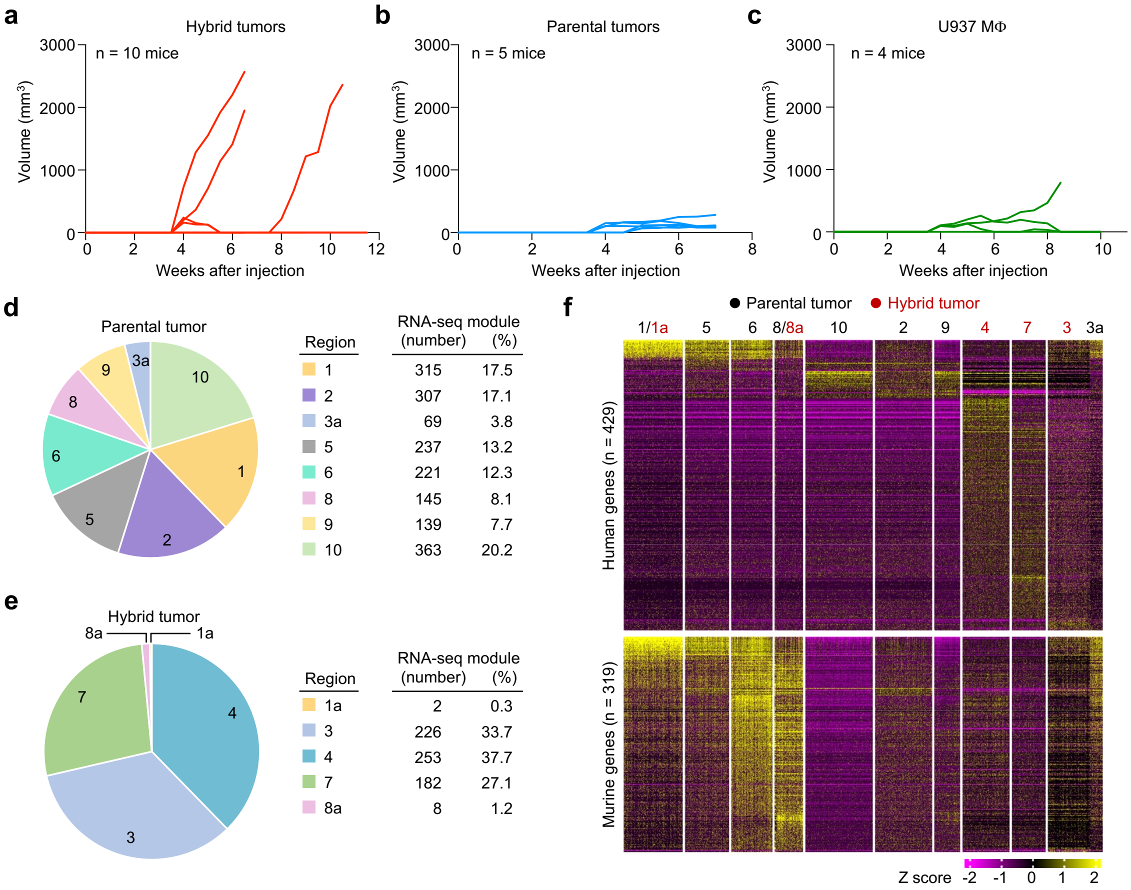

Supplementary Fig. 2. Spatial transcriptomics analysis of parental and hybrid tumors in murine xenografts. Related to Fig. 2

a-c Growth curves of xenograft tumors. Nu/Nu mice were subcutaneously inoculated with a 7-day co-culture mixture of U937 macrophages and C4-2 cells, which contained ~50% putative THCs (a), C4-2 cells (b), or U937 macrophages (c). **d**, **e** Pie charts showing the proportions of each region in parental (d) or hybrid tumor (e) identified in Fig. 2b. The numbers of RNA-seq modules (spatial spots) captured in parental (d) or hybrid tumor (e) were listed on the *right*. **f** Heatmap showing the differential expression patterns of human (*upper*) or murine (*lower*) genes in each region. Source data for a-c are provided as a Source Data file.

Supplementary Fig. 3 (Related to Fig. 2)

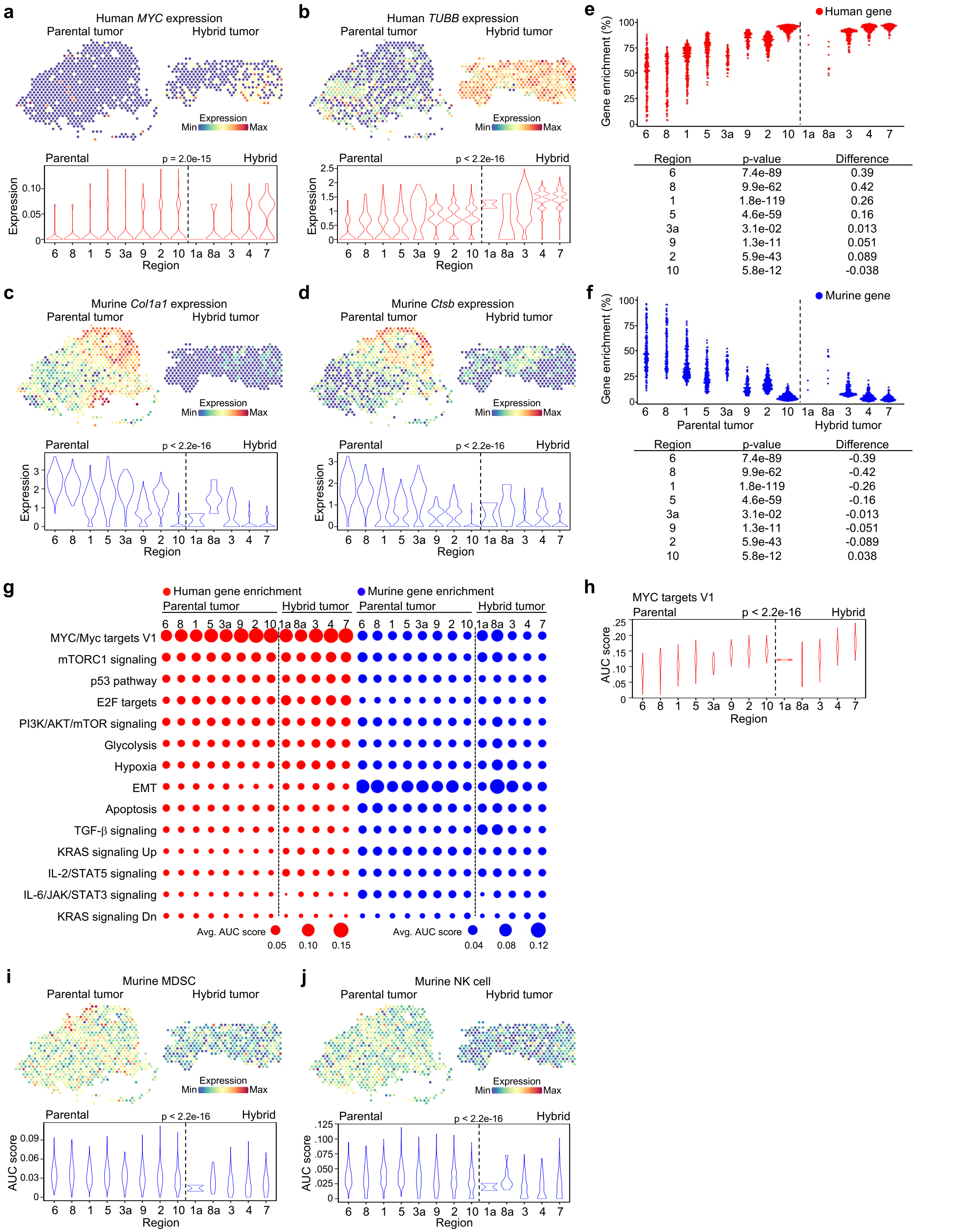

Supplementary Fig. 3. Hybrid tumor had higher human gene expressions and lower murine gene expressions. Related to Fig. 2

a-d *Upper*: representative spatial heatmap showing the expression of human *MYC* (a), human *TUBB* (b), murine *Col1a1* (c), or murine *Ctsb* (d) in the parental tumor regions 1, 5, and 6, and hybrid tumor regions 4 and 7. Lower: violin plots showing the expression of the four aforementioned genes in each region. **e**, **f** Upper: beeswarm plot showing the enrichment scores of human (e) or murine (f) genes. *Lower*: differences in gene enrichment scores between the hybrid tumor regions (1a, 8a, 3, 4, and 7) and each parental region. **g** Relative enrichment of 14 human (red) or murine (blue) oncogenic MSigDB genesets across different regions in the parental and hybrid tumors analyzed by AUCell analysis. The size of each dot represents the average gene signature score over all spots in each region. **h,** Violin plots showing the mean enrichment score of the MYC targets V1 geneset in each region. **i**, **j** *Upper*: representative spatial heatmap showing the mean expression of murine myeloid-derived suppressor cells (MDSCs) (i) or natural killer (NK) cell (j) genesets in the parental tumor regions 1, 5, and 6, and hybrid tumor regions 4 and 7. *Lower*: violin plots showing the mean enrichment scores of the aforementioned genesets in each region. The hybrid tumor had less MDSC or NK cell gene expression, suggesting that less infiltration of these cells in the hybrid tumor compared with the parental tumor. *P*-values were determined using a two-sided unpaired *t*-test.

Supplementary Fig. 4 (Related to Fig 3)

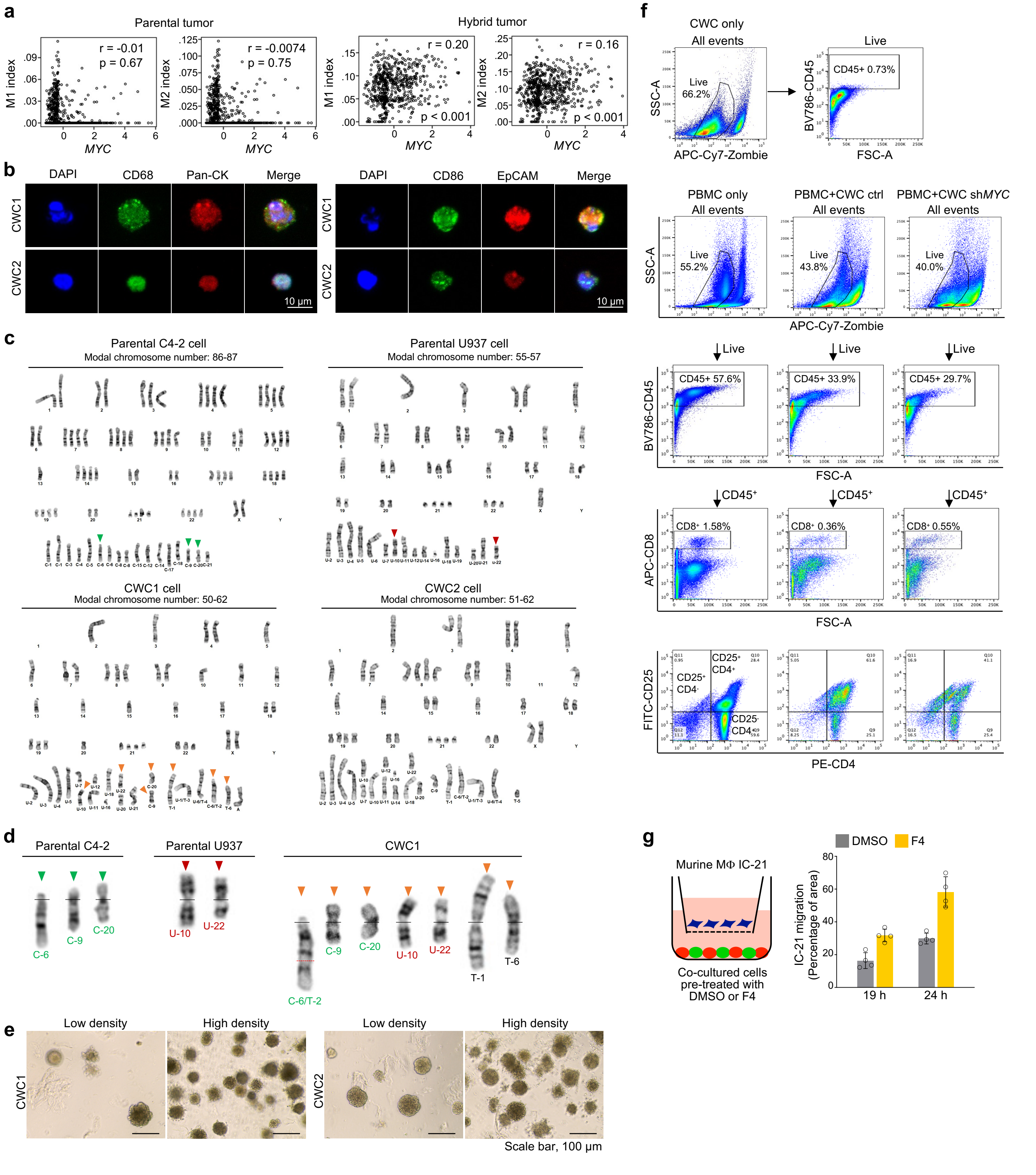

Supplementary Fig. 4. CWC cells derived from hybrid tumors acquired signatures from parental macrophages and cancer cells. Related to Fig. 3

a Scatter plots showing the correlation between *MYC* and human M1- or M2-like macrophage index in parental (*left*) or hybrid tumor (*right*). Each scatter dot represented a spot in spatial transcriptomics assay shown in Fig 2. **b** Representative immunofluorescent images of macrophage marker CD68 and epithelial marker pan-cytokeratin (pan-CK) or CD86 and EpCAM in two CWC lines derived from xenograft hybrid tumors, CWC1 and CWC2. CWC cells expressed both macrophage and epithelial markers. **c** Karyotypes of a parental C4-2 cell, a parental U937 cell, and two CWC cell lines. Complex karyotypes were presented in CWC cells (n = 8 analyzed) with a modal chromosome number ranging from 50 to 62, while C4-2 cells (n = 4) had 86-87 and U937 cells (n = 4) had 55-57 chromosomes. **d** Representative marker chromosomes (G-banding patterns) of parental C4-2 cell, parental U937 cell, and CWC cell. New marker chromosomes were observed in CWC cell (e.g., T-1, T-2, and T-6), with T-2 possibly containing part of an inverted C-6. **e** CWC1 and CWC2 cells grew in Matrigel for 8 days. Scale bar, 100 μm. **f** Flow cytometry gating strategy for cell type identification in Fig. 3h. Live cells with lower Zombie dye staining were firstly gated for further analysis. The $CD45^+$ population was then identified. The $CD45^+/CD8^+$ cells were designated as cytotoxic T cells, the $CD45^+/CD25^+/CD4^+$ cells were designated as regulatory T cells cells, and $CD45^+/CD25^-/CD4^+$ cells were designated as naïve T cells. **g** *Left*: schematic diagram of the trans-well assay. Co-cultured cells pre-treated with DMSO or c-Myc inhibitor 10058-F4 were seeded in the lower chamber and mouse IC-21 macrophages were seeded in the upper chamber. *Right*: bar graph showing the area percentage of mouse macrophages migrating to the lower chamber. Data are the mean ± SD of four technical repeats of each time point. *P*-values were determined using a Spearman's rank correlation (a). Source data for c, g are provided as a Source Data file.

Supplementary Fig. 5 (Related to Fig 5)

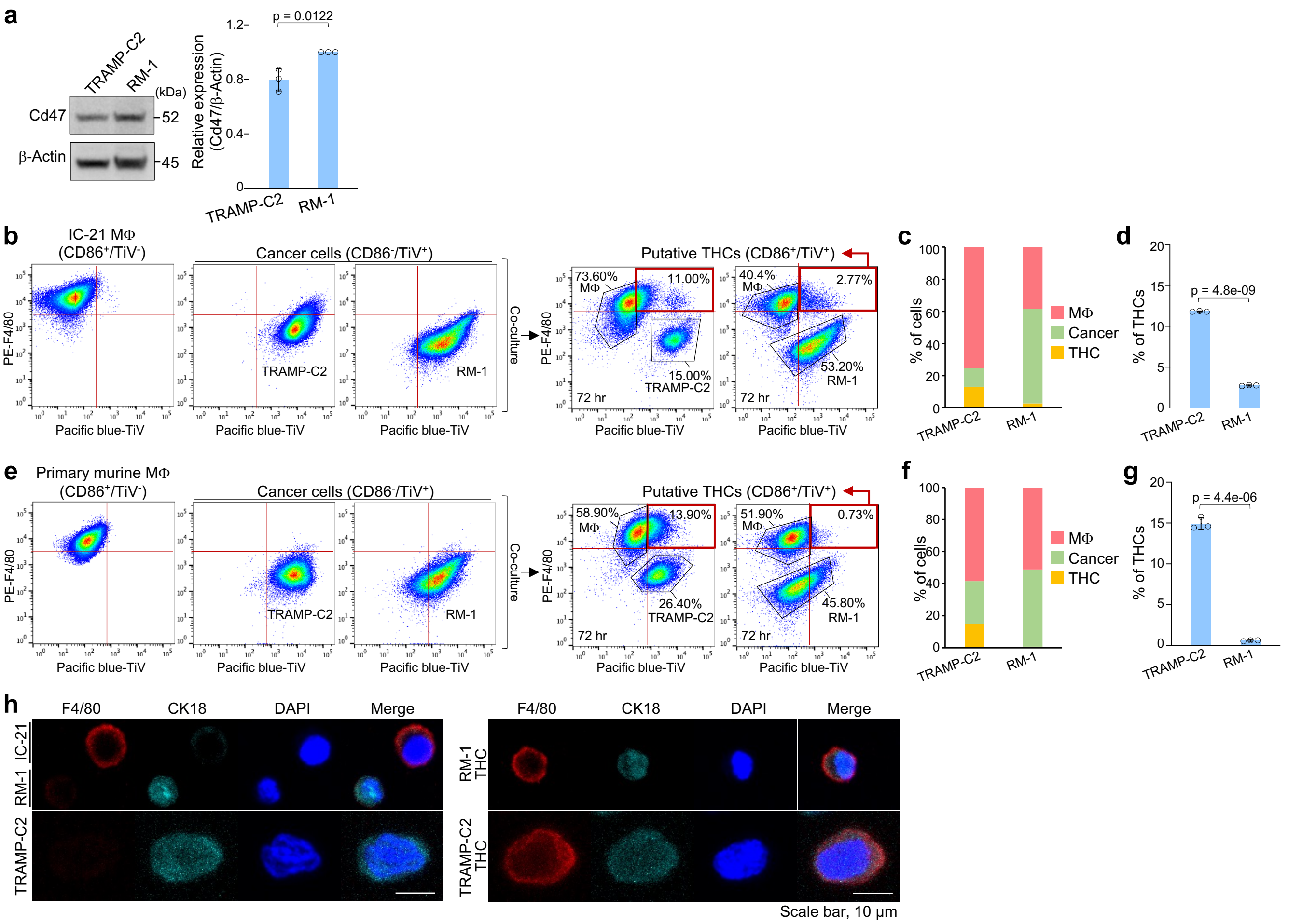

Supplementary Fig. 5. Murine THCs generated through co-culture. Related to Fig. 5
a Western blotting analysis of Cd47 expression levels in two mouse prostate cancer cell lines TRAPM-C2 and RM-1. **b-g** Flow cytometry analysis of TiV-labeled TRAMP-C2 or RM-1 cells co-culture with IC-21 cells (b-d) or murine primary macrophages (e-g) at 72 hours. Compared to RM-1 cells, more TRAMP-C2 cells were engulfed by IC-21 or primary macrophages, suggesting more THCs can be formed in low Cd47-expressing cells. The proportion of each cell type in the co-cultured models was shown in c, d, f, and g. Note that there was no significant difference in the overall frequency of THC formation between IC-21 and primary macrophages. However, the frequency may vary in a context-dependent manner as primary macrophages tended to cause a lower efficiency than U937 macrophages in human models (see also Fig. 1e). **h** Representative immunofluorescence images of murine macrophage marker F4/80 and epithelial marker cytokeratin 18 (CK18) in IC-21 and RM-1 or TRAPM-C2 co-culture model. THCs (*right*) had both F4/80 and CK18 expressions, while the parental cells (*left*) only had either F4/80 or CK18 expression. Scale bar, 10 μm. Data are the mean ± SD of three independent experiments. *P*-values were determined using a two-sided unpaired *t*-test. Source data for a, c, d, f, g are provided as a Source Data file.

Supplementary Fig. 6 (Related to Fig 5)

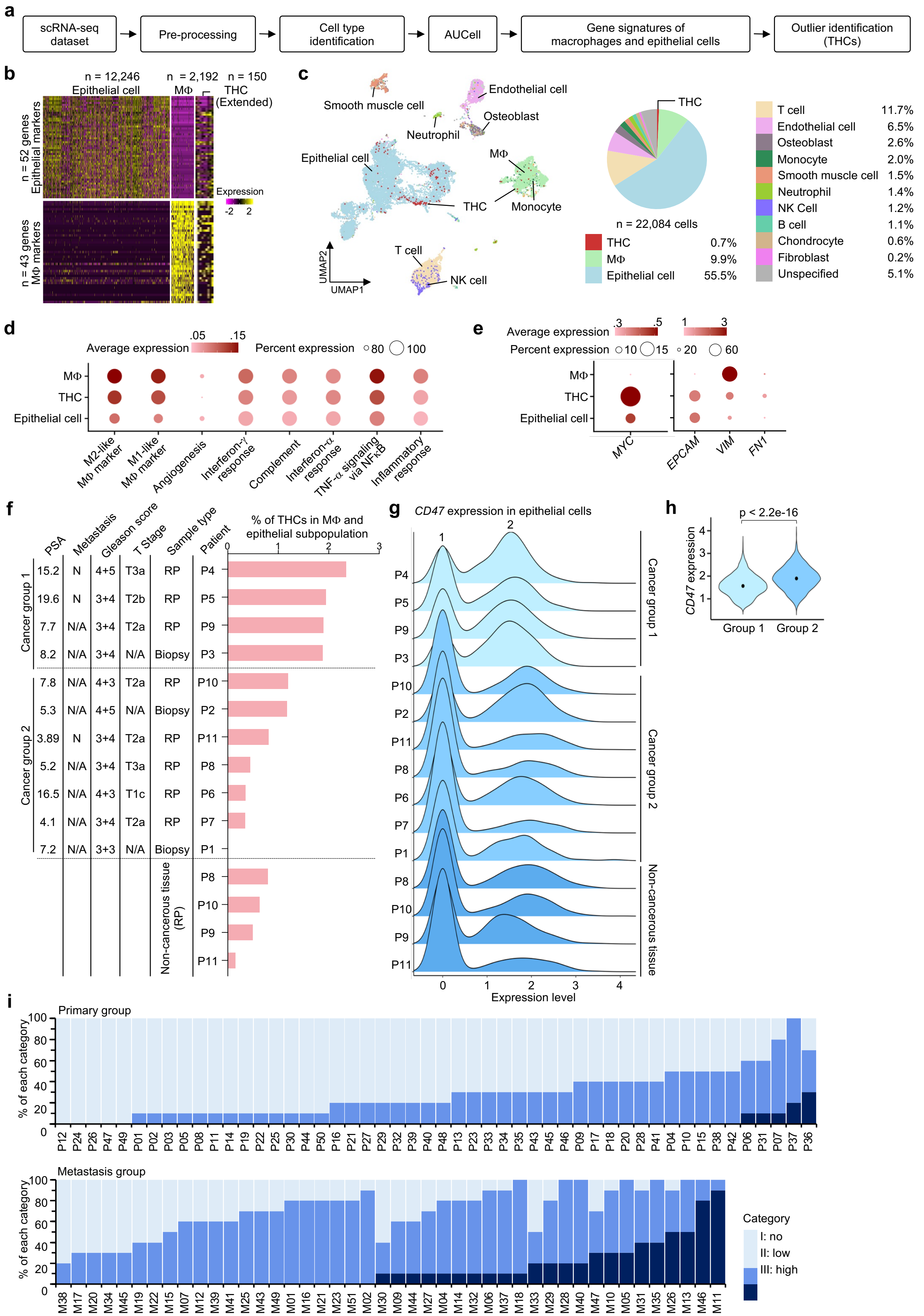

Supplementary Fig. 6. THCs found in human prostate cancer patients through *in silico* analysis and immunohistochemistry staining. Related to Fig. 5

a Workflow of THC identification from a published single-cell RNA-seq dataset of 11 cancerous tissues and 4 adjacent non-cancerous tissues[1]. Putative THCs were identified based on the co-expression of epithelial (n = 52) and macrophage (n = 43) genes. **b** Heatmaps showing the expression of epithelial and macrophage genes in the epithelial cells, macrophages, and THCs population. ***c*** *Left*: UMAP depicting different cell types identified in the cancerous tissues and adjacent non-cancerous tissues. Note that the putative THCs were embedded in either macrophage or epithelial population. *Right*: pie chart showing the proportion of each cell type. **d**, **e** AUCell analysis showing average expression level and the percentage of expressed cells of the eight immune pathways (d) or selected genes - *MYC*, *EPCAM*, *VIM*, and *FN1* (e) in macrophages, THCs, and epithelial cells. THCs had a higher expression of immune markers, *MYC*, *EpCAM*, *VIM*, and *FN1* than epithelial cells. **f** Percentage of putative THCs and the corresponding clinicopathological information of each patient (P1-11). Cancerous tissues were divided into two groups based on the percentage of THCs. Note that the frequencies of THCs in Group 1 were higher than that of non-cancerous tissues. **g** Ridgeline plots displayed two peaks, one with and the other without detectable *CD47*, in epithelial cells of each tissue sample. **h** Violin plots showing *CD47* expression of the epithelial cells (peak 2) in Group 1 and Group 2 cancerous tissues. Higher frequencies of THC formation tended to occur in primary tumors with lower *CD47* levels (Group 1) than tumors with higher levels (Group 2) in the *CD47*-expressing subpopulations. The four non-cancerous tissues all had comparable expression levels of *CD47*, and yet the lower incidence of THC formation than that of Group 1 tumors. One probable explanation is fewer macrophage infiltrations in non-cancerous tissues than in adjacent and tumor sites (see Fig. 5b-*middle-right*). **i** Bar graph depicting the proportion of no-, low-, or high-THC categories in each primary (*upper*, n = 50) or metastatic (*lower*, n = 44) tumor section (see Fig. 5l). *P*-values were determined using a two-tailed unpaired *t*-test.

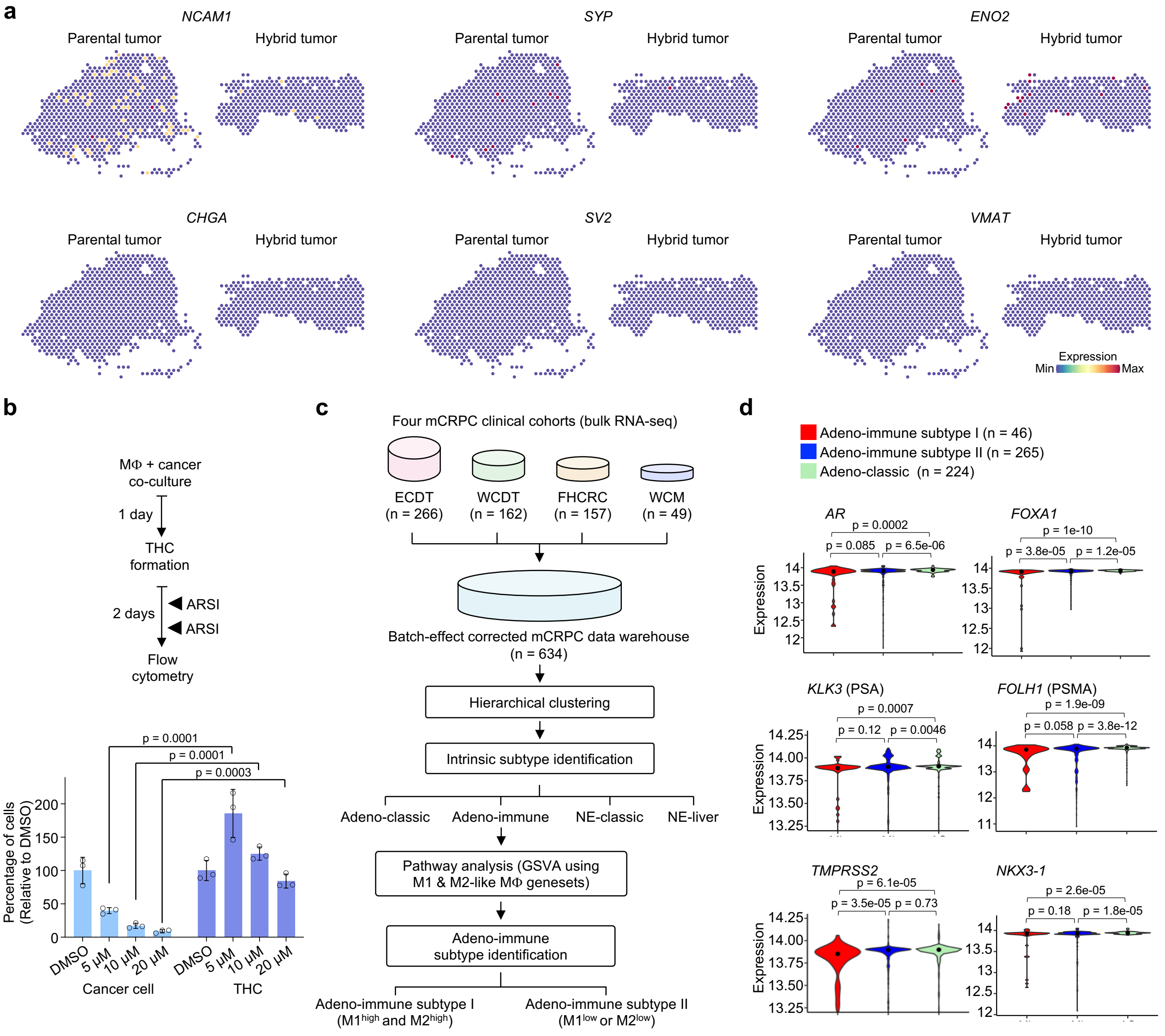

Supplementary Fig. 7. Low expression of neuroendocrine (NE) genes in the hybrid tumor and low expression of androgen receptor (AR)-related genes in Adeno-immune subtype I prostate cancer. Related to Fig. 6

a Representative spatial heatmap depicting the expression of six neuroendocrine (NE) genes in parental tumor regions 1, 5, and 6, and hybrid tumor regions 4 and 7. **b** Flow cytometry analysis of the effects of AR signaling inhibitor (ARSI) enzalutamide on co-culture cells. *Upper*: workflow of co-culture experiment and ARSI treatments. U937 macrophages and C4-2 cancer cells were co-cultured for 24 hours to form THCs, followed by 48 hours of DMSO or 5-20 μM enzalutamide treatments. *Lower*: relative population size of parental cancer cells or THCs in co-culture after DMSO or ARSI stimulation. Parental cancer cells were more sensitive to enzalutamide treatment compared to THCs. **c** Workflow of *in silico* analysis from four published RNA-seq datasets. Four subtypes of metastatic castration-resistant prostate cancer (mCRPC) were identified and described by Feng *et al*[2]. The Adeno-immune group was further divided into subtype I and subtype II based on the expression of M1- and M2-like macrophage genesets described in Supplementary Table 1. **d** Violin plots depicting the expression of *AR*, AR cooperating gene *FOXA1*, and AR-target genes *KLK3*, *FOLH1*, *TMPRSS2*, and *NKX3-1* in Adeno-immune subtype I, subtype II, and Adeno-classic groups. Data are the mean ± SD of three independent experiments. *P*-values were determined using a two-sided unpaired *t*-test. Source data for b are provided as a Source Data file.

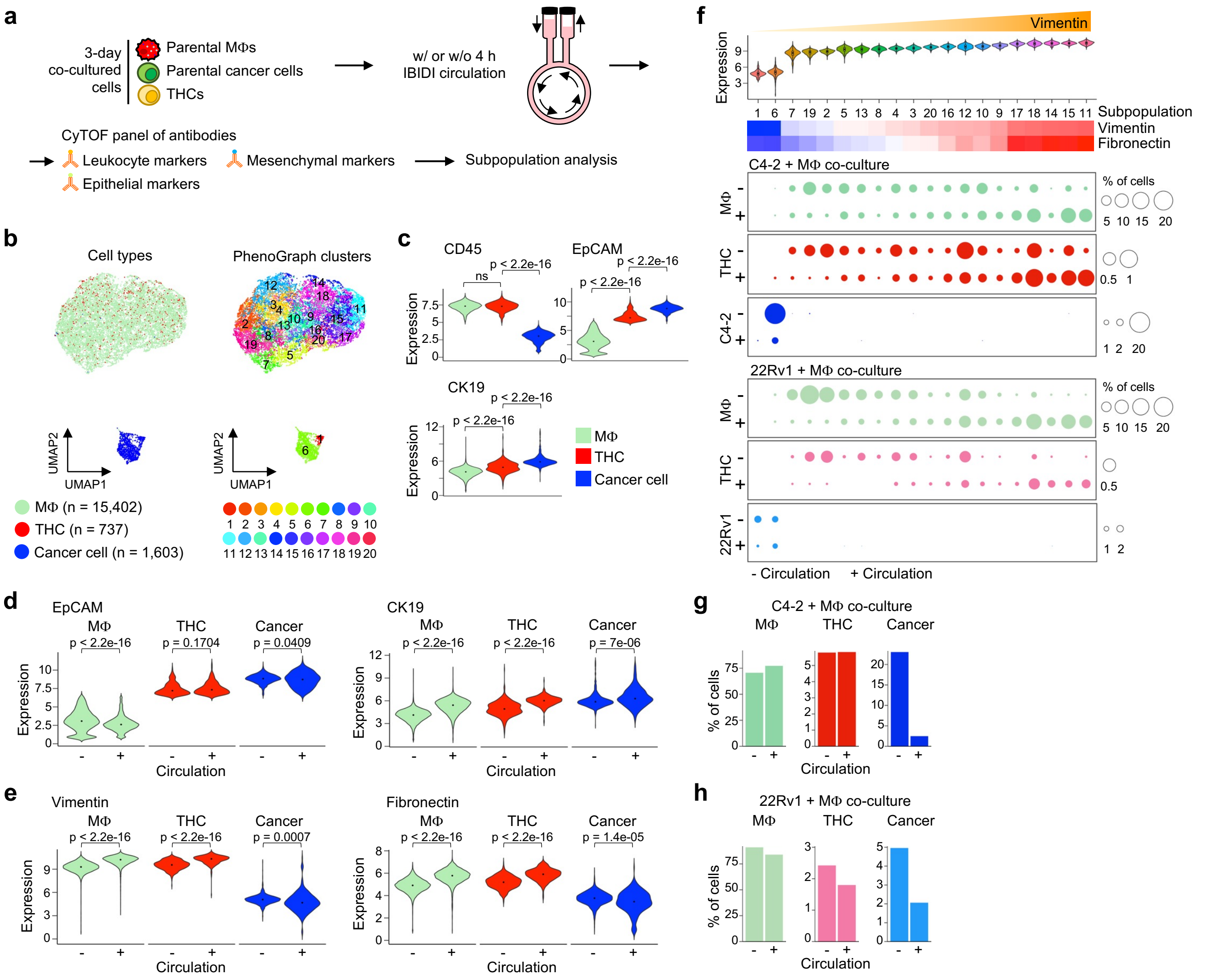

Supplementary Fig. 8. THCs gained more mesenchymal features to enhance adaption to circulation stress. Related to Fig. 7

a Schematic diagram depicting the process of characterizing THCs in an *in vitro* circulation test. U937 macrophages and C4-2 or 22Rv1 cells were co-cultured for 3 days and then subjected to circulation stress through the ibidi microfluidic system in two independent experiments. The cell type and protein expressions were analyzed by CyTOF. **b** *Left*: UMAP depicting THCs (CD45$^+$/EpCAM$^+$), parental macrophages (CD45$^+$/EpCAM$^-$), and parental cancer cells (CD45$^-$/EpCAM$^+$) in the co-culture. *Right*: UMAP depicting 20 subpopulations of the co-cultured cells before and after ibidi circulation stress stimulation. **c** Violin plots depicting the expressions of CD45, EpCAM, and CK19 in the parental cells and THCs before ibidi circulation stress stimulation. **d**, **e** Violin plots depicting the expressions of epithelial (d) and mesenchymal markers (e) in the parental cells and THCs without (-) or with (+) ibidi circulation stress stimulation. Expression levels of Vimentin and Fibronectin were elevated in parental macrophages and THCs after circulating stimulation. **f** The 20 subpopulations in (b) were aligned by the increased expression levels of Vimentin. *Upper*: Vimentin expression in the 20 subpopulations. Middle: heatmap showing the expression of Vimentin. *Lower*: circle plots representing the size of each subpopulation without (-) or with (+) ibidi circulation stress test. High Vimentin-expressing subpopulations (#9, 17, 18, 14, 15, and 11) of macrophages or THCs were shown to expand after the circulation stress test. **g**, **h** Percentage of parental cells and THCs without (-) and with (+) ibidi circulation stress test. The proportions of parental macrophages and THCs did not change much after the circulation test, but those of parental C4-2 (g) or 22Rv1 (h) cancer cells showed a dramatic decrease. *P*-values were determined using a two-sided unpaired *t*-test.

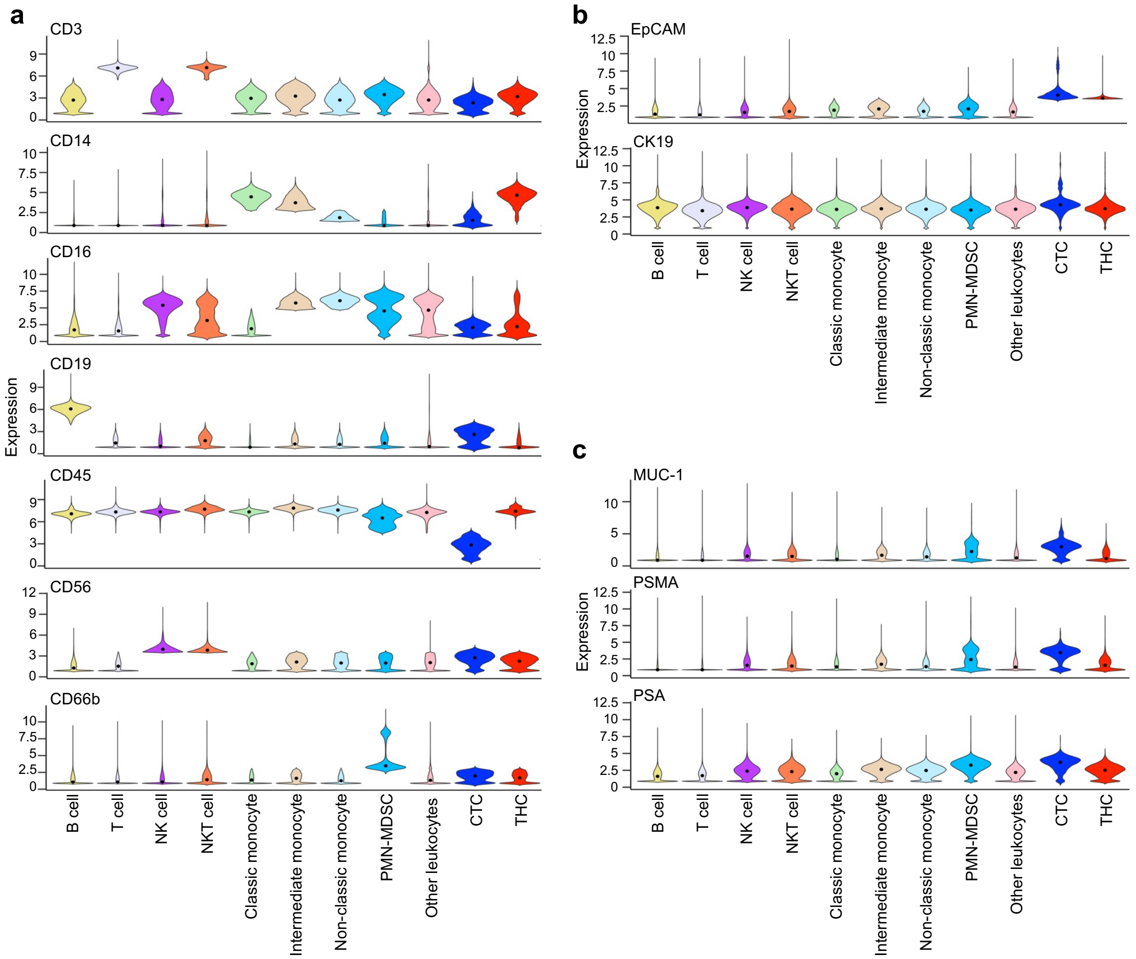

Supplementary Figure 9. Expression levels of immune, epithelial, and AR-related markers in each cell type in patients' PBMCs. Related to Fig. 8

a-c Violin plots showing the expression of immune (a), epithelial (b), and AR-related (c) markers in each cell type shown in Fig. 8c. PBMCs were isolated from 16 prostate cancer patients.

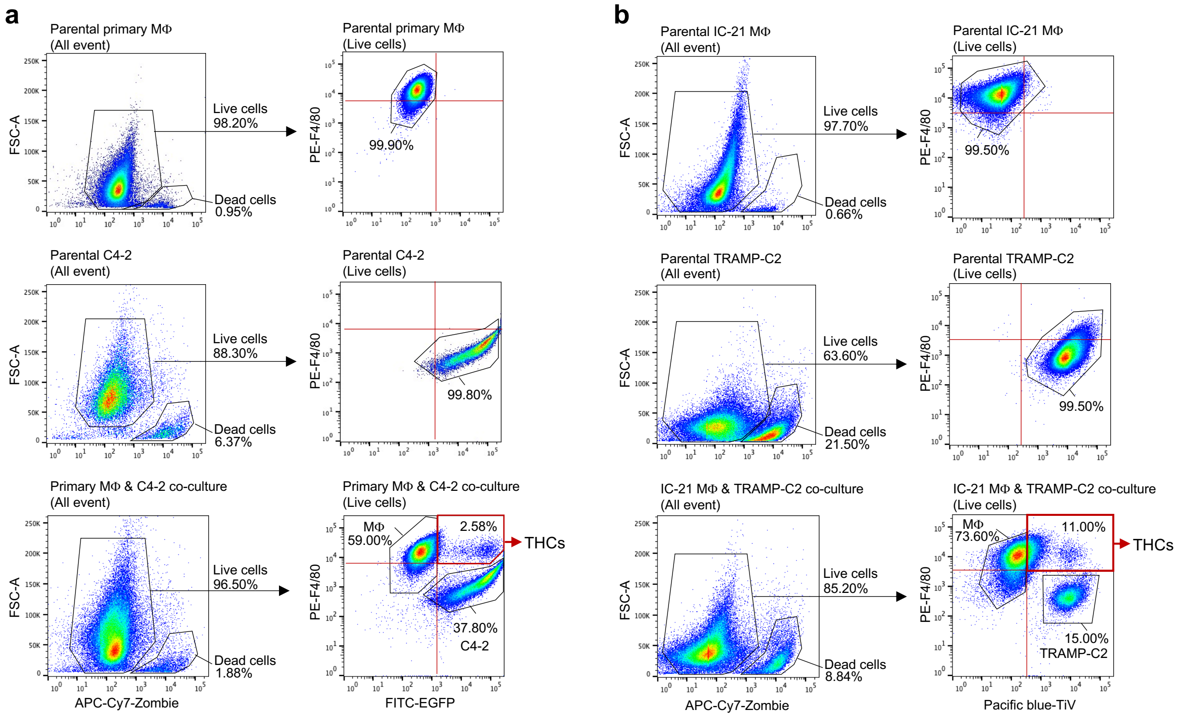

Supplementary Fig. 10. Representative flow cytometry gating strategies.

a Representative gating strategies of THC identification using human cell co-culture model; related to Fig. 1d-1j, Fig. 3a, b, Supplementary Fig. 1d-k, m, and Supplementary Fig. 7b. Live cells with lower Zombie dye staining were firstly identified for further analysis. Then, the CD86 and EGFP expression profiles of the parental cells were checked. After co-culturing, the CD86$^+$/EGFP$^+$ population in live cells was designated as THCs. **b** Representative gating strategies of THC identification using mouse cell co-culture model; related to Supplementary Fig. 5b-g. Live cells with lower Zombie dye staining were firstly identified for further analysis. Then, the F4/80 and TiV expression profiles of the parental cells were checked. After co-culturing, the F4/80$^+$/TiV$^+$ population in live cells was then designated as THCs.

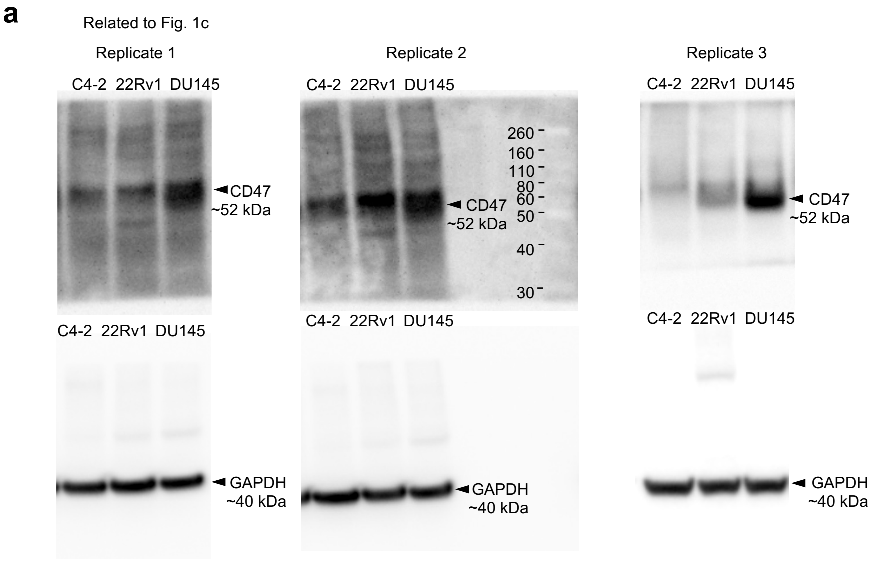

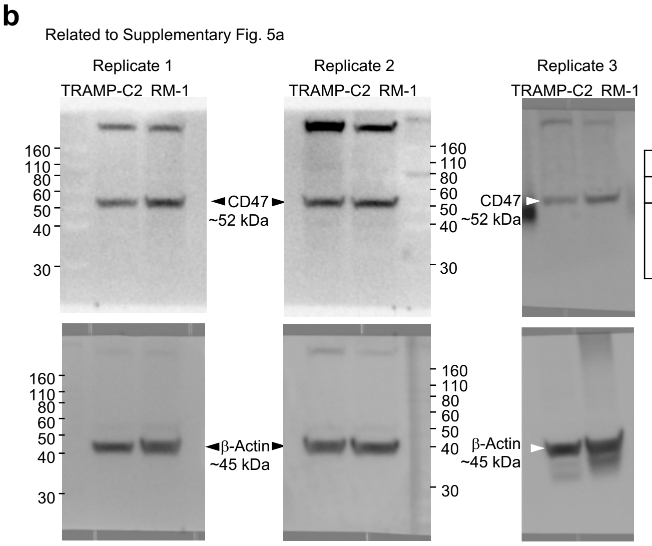

	Replicate 1		Replicate 2		Replicate 3	
	TRAMP-C2	RM-1	TRAMP-C2	RM-1	TRAMP-C2	RM-1
CD47 (RawIntDen)/ β-Actin (RawIntDen)	0.73	0.83	0.51	0.72	0.33	0.41

Supplementary Fig. 11. Uncropped images of Western blot in Figures.
a Western blot of CD47 and the corresponding internal control in Fig. 1c. Note that the GAPDH was blotted after striping the CD47 signal in replicates 1 and 2. In replicate 3, CD47 and GAPDH were probed in two Western blots of the same replicated samples. **b** Western blot of CD47 and the corresponding internal control in Supplementary Fig. 5a. Note that the β-Actin was blotted after stripping the CD47 signal in replicates 1, 2, and 3. The raw integrated density of each signal was calculated through Fiji Image J, and the ratio of CD47 to its β-Actin was listed in the right table.

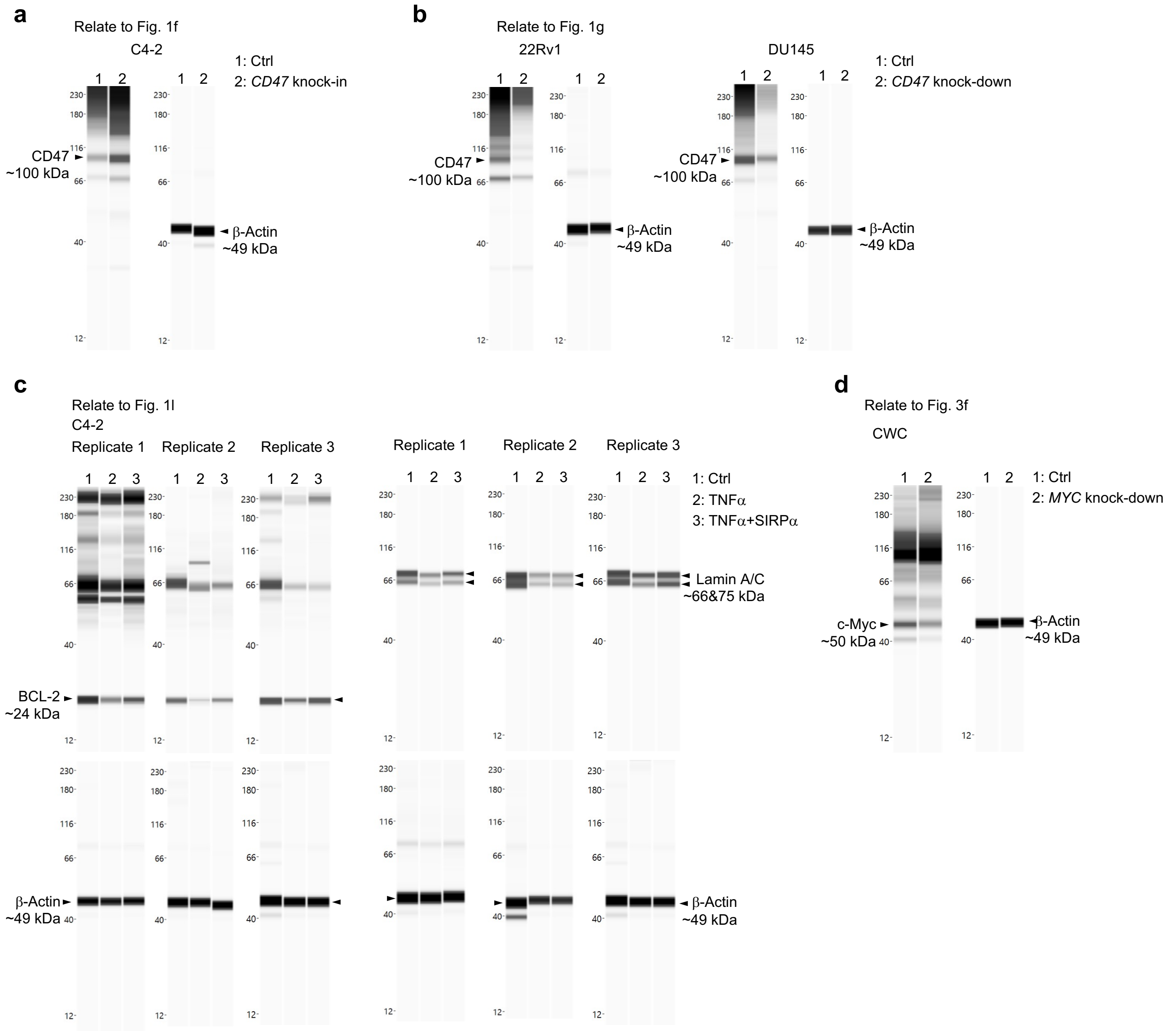

Supplementary Fig. 12. Uncropped images of Capillary Western immunoassay (WES) in Figures.

a, **b** WES of CD47 and β-Actin in CD47 knock-in or -down cells for Fig. 1f (a) or 1g (b). A specific band of CD47 was detected at ~110 kDa, similar to the manufacturer's data (Novus Biologicals, NBP3-14713). **c** WES of BCL-2, Lamin A/C, and the corresponding β-Actin in Fig. 1l. Although there were unspecified bands shown at high molecular weights, the specific band of BCL-2 was detected at ~24 kDa based on the manufacturer's data. The corresponding β-Actin bands are shown on the lower panel. Note that replicate 3 of BCL-2 and Lamin A/C shared the same internal control. **d** The c-Myc antibody (R&D System, MAB3696) detected a key band at ~50 kDa, showing a decreased intensity in CWC cells carrying *MYC* shRNA relative to CWC cells carrying a control vector. Unspecified bands at high molecular weights were noted, displaying no overt difference in blotting intensities. The corresponding β-Actin band is shown on the right panel.

Supplementary Table 1. Markers selected for pathway analysis. Related to Fig. 2, Fig. 6, Supplementary Fig. 3, and Supplementary Fig. 6

Pathway	Gene sets	References (PMID)
M1-like macrophage marker (human)	*CD80, CD86, CD36, CD68, CXCL9, CXCL10, FCGR2B, IFNG, IFNGR1, IL1B, IL6, IL12B, IL15, IL17A, IL18, IL23A, IRF5, HLA-A, HLA-B, iNOS, STAT1, TNF, CD38, HLA-DR, HLA-C*<br>Number of genes = 25	20856220[3], 31530089[4], 23766387[5], 22378047[6], 26699615[7], 31178859[8], 32039007[9], 34789834[10]
M1-like macrophage marker (mouse)	*Cd80, Cd86, Cd36, Cd68, Cxcl9, Cxcl10, Fcgr2b, Ifng, Ifngr1, Il1b, Il6, Il12b, Il15, Il17a, Il18, Il23a, Irf5, H2-D1, Nos2, Stat1, Tnf, Cd38, H2-Eb1, H2-T3, H2-K1*<br>Number of genes = 25	20856220[3], 31530089[4], 23766387[5], 22378047[6], 26699615[7], 31178859[8], 32039007[9], 34789834[10]
M2-like macrophage marker (human)	*CSF1R, MRC1, PPARG, ARG1, CD163, CLEC10A, CLEC7A, PDCD1LG2, RETNLB, CCL22, IL10, IL4, IRF4, PDGFB, HLA-A, HLA-B, STAT3, HLA-E, HLA-C, CCL17, CCL24*<br>Number of genes = 21	20856220[3], 31530089[4], 23766387[5], 22378047[6], 31178859[8], 32039007[9], 34789834[10], 31554795[11]
M2-like macrophage marker (mouse)	*Csf1r, Mrc1, Pparg, Arg1, Cd163, Clec10a, Pdcd1lg2, Retnla, Ccl22, Il10, Il4, Irf4, Pdgfb, Chil3, H2-D1, Stat3, H2-T3, H2-K1*<br>Number of genes = 18	20856220[3], 31530089[4], 23766387[5], 22378047[6], 31178859[8], 32039007[9], 34789834[10], 31554795[11]
NK cell marker (mouse)	*Fcgr4, Ncam1, Klrd1, Kir3dl2, Ncr1, Klrb1c, Itga1, Cxcr6, Itgax, Itgae, Kit, Ifngr1, Il2rb, Il7r, Fcgr3, Cd160, Klrb1, Klrb1b, Klrb1c, Cxcr2, Cxcr3, Ccr6, Cd1d1, Cd2, Il12rb1, Pecam1, Klrk1, Slamf7, Ncr1, Cd34, Cd44, Ptprc, Ptprc, Ptprc, Ptprc, Itga2, B3gat1, Cd69, Plaur, Thy1, Klrd1, Cd96, Cx3cr1, Flt3, Klrg1, Zbtb16, Rorc, Ly6a, Il1rl1*<br>Number of genes = 49	30150991[12], 25711213[13], 35652109[14], 31477722[15], 25312647[16]
MDSC marker (mouse)	*Itgam, Ly6g, Il4ra, Csf1r, Arg1, Csf1r, Kit, Ifngr1, Itgam, Itgax, Il2rb, Il3ra, Il6ra, Il7r, Anpep, Cd14, Thbd, Cd40lg, Fcgr3, Siglecf, Cxcr2, Cd19, Ccr2, Ccr3, Ccr4, Cd1d1, Enpp3, Mrc1, Il18r1, Fcer2a, Cd24a, Pdcd1lg2, Tlr2, Tlr4, Ptgdr2, Pecam1, Cdh1, Cd33, Cd34, Cr2, Tnfrsf18, Cd38, Cd40, Itga2b, Spn, Cd44, Ptprc, Ptprc, Itga2, Sell, Fcgr1, Cd68, Cd69, Cd80, Cd84, Cd86, Plaur, Cd9, Thy1, Cd93, Cx3cr1, Adgre1, Fcer1a, Flt3, Il1r1, Il1r2, Il1r1, Il1r2, Nos2, Ly6c1, Ly6g, Ly6a, Siglecf*<br>Number of genes = 73	33979585[17], 24060865[18], 25504825[19], 27057424[20], 33526920[21]
MYC targets v1	Molecular Signatures Database (MSigDB) HALLMARK_MYC_TARGETS_V1<br>Number of genes = 200	16199517[22], 21546393[23], 26771021[24]
mTORC1 signaling	Molecular Signatures Database (MSigDB) HALLMARK_MTORC1_SIGNALING<br>Number of genes = 200	16199517[22], 21546393[23], 26771021[24]
p53 pathway	Molecular Signatures Database (MSigDB) HALLMARK_P53_PATHWAY<br>Number of genes = 200	16199517[22], 21546393[23], 26771021[24]
E2F targets	Molecular Signatures Database (MSigDB) HALLMARK_E2F_TARGETS<br>Number of genes = 200	16199517[22], 21546393[23], 26771021[24]
PI3K/AKT/mTOR signaling	Molecular Signatures Database (MSigDB) HALLMARK_PI3K_AKT_MTOR_SIGNALING<br>Number of genes = 105	16199517[22], 21546393[23], 26771021[24]
Glycolysis	Molecular Signatures Database (MSigDB) HALLMARK_GLYCOLYSIS<br>Number of genes = 200	16199517[22], 21546393[23], 26771021[24]
Hypoxia	Molecular Signatures Database (MSigDB) HALLMARK_HYPOXIA<br>Number of genes = 200	16199517[22], 21546393[23], 26771021[24]
EMT	Molecular Signatures Database (MSigDB) HALLMARK_EPITHELIAL_MESENCHYMAL_TRANSITION<br>Number of genes = 200	16199517[22], 21546393[23], 26771021[24]
Apoptosis	Molecular Signatures Database (MSigDB) HALLMARK_APOPTOSIS<br>Number of genes = 161	16199517[22], 21546393[23], 26771021[24]
TGF-β signaling	Molecular Signatures Database (MSigDB) HALLMARK_TGF_BETA_SIGNALING<br>Number of genes = 54	16199517[22], 21546393[23], 26771021[24]
KRAS signaling Up	Molecular Signatures Database (MSigDB) HALLMARK_KRAS_SIGNALING_UP<br>Number of genes = 200	16199517[22], 21546393[23], 26771021[24]
IL-2/STAT5 signaling	Molecular Signatures Database (MSigDB) HALLMARK_IL2_STAT5_SIGNALING<br>Number of genes = 199	16199517[22], 21546393[23], 26771021[24]
IL-6/JAK/STAT3 signaling	Molecular Signatures Database (MSigDB) HALLMARK_IL6_JAK_STAT3_SIGNALING<br>Number of genes = 87	16199517[22], 21546393[23], 26771021[24]
KRAS signaling Dn	Molecular Signatures Database (MSigDB) HALLMARK_KRAS_SIGNALING_DN<br>Number of genes = 200	16199517[22], 21546393[23], 26771021[24]
Angiogenesis	Molecular Signatures Database (MSigDB) HALLMARK_ANGIOGENESIS<br>Number of genes = 36	16199517[22], 21546393[23], 26771021[24]
Interferon-γ response	Molecular Signatures Database (MSigDB) HALLMARK_INTERFERON_GAMMA_RESPONSE<br>Number of genes = 200	16199517[22], 21546393[23], 26771021[24]

Complement	Molecular Signatures Database (MSigDB)<br>HALLMARK_COMPLEMENT<br>Number of genes = 200	16199517[22], 21546393[23], 26771021[24]
Interferon-α response	Molecular Signatures Database (MSigDB)<br>HALLMARK_INTERFERON_ALPHA_RESPONSE<br>Number of genes = 97	16199517[22], 21546393[23], 26771021[24]
TNF-α signaling via NFκB	Molecular Signatures Database (MSigDB)<br>HALLMARK_TNFA_SIGNALING_VIA_NFKB<br>Number of genes = 200	16199517[22], 21546393[23], 26771021[24]
Inflammatory response	Molecular Signatures Database (MSigDB)<br>HALLMARK_INFLAMMATORY_RESPONSE<br>Number of genes = 200	16199517[22], 21546393[23], 26771021[24]

Supplementary Table 2. Sequences of oligonucleotides. Related to Fig. 3

ChIP-qPCR

Gene	Forward primer 5'-3'	Reversed primer 5'-3'
IRF5	AGTGGTTTGAGCACAGT	GCCTCTGTGCCCCTGTC
CD38	CAGCACTCCGATTCTTTTTG	CGCCTTATCATCTAAGTAATGTCT
CD163	CTAAATCTTCTTGTATTATTCCCTAGAAA	GTTGACTCCGCCTCCAT
HLA-A	GTCCTTGGCAGTTGTCT	GTATCCACTTCTAAGTGTTGTGTA
HLA-E	GTTTCCCGTTCCTCTCGTAA	GACGCTGATTGGCTTCTCTA
PPARG	TTTGGACAGGTCACGATGGA	ACACCGAGGGACTCAGA
CD86	GATGGGGTAACTGTGGC	TGGAGAAACCTTCTGGCG
CD80	TGTGAGGTGGCTAGGGATT	GGGGCTGTGTCCTGGTA
MRC1	TCCCAGGCTGTTTCACT	AGAAGAGCAGACGCAGA

RT-qPCR

Gene	Forward primer 5'-3'	Reversed primer 5'-3'
MYC	CTGAGGCACACAAAGACT	GGCTTGGACAGGTTAGGA
IRF5	GGGAAATTCAGCCATGAGC	CTTTTGTTGCCAGAGGAAAT
CD38	ACTGGATTACTGGTTGCTC	ATGATGGTGGGTGAAAGTT
CD163	GAGGAACAGACAAGGAGC	AGCCATTATTACACACCGT
HLA-A	AAGCCAATCAGTGTCGT	GTAGCAGGAGGAGGGTT
HLA-E	GCAGCACACGAAGTCAA	AAGTTTACCTCAGATCAGCC
PPARG	AGCAGAGCAAAGAGGTG	TGGTCGTTCAAGTCAAGATT
CD86	GAACCAAGCAAGAGCACT	CTCCCCGTACCTCCTAAG
CD80	CTCACTATGCTGCTTCACAA	CAATACGGGAAACACTGCTA
MRC1	GAAAGCCAGGGTGTGTTG	GGTGGGTGGGTTACTCC
UBB	GGTCCTGCGTCTGAGAGGT	GGCCTTCACATTTTCGATGGT

shRNA

Target gene	TRC clone ID	Target sequence 5'-3'
CD47	TRCN0000007836 (Sigma-Aldrich)	GCTTCCAATCAGAAGACTATA
MYC	TRCN0000174055 (Sigma-Aldrich)	CCTGAGACAGATCAGCAACAA

Supplementary Table 3. CyTOF antibody panel for metastatic human hybrid tumor and murine peripheral blood mononuclear cells (PBMCs). Related to Fig. 4 and Fig. 7

Tag	Antigen	Clone	Marker type	Reactivity	Vendor	Cat. No.	RRID	Amount (μl)*
89Y	CD45	30-F11	Leukocyte	Mouse	Fluidigm	3089005B	AB_2651152	1
146Nd	F4/80	BM8	Leukocyte/ macrophage	Mouse	Fluidigm	3146008B	AB_2895117	1
159Tb	Ly-6G/Ly-6C (Gr-1)	RB6-8C5	Leukocyte/MDSC	Mouse	R&D Systems	MAB1037	AB_2232806	1
116Cd	TNF-alpha	Polyclonal	M1-like macrophage	Human/ Mouse	R&D Systems	AF-410-NA	AB_354479	1
144Nd	CXCL9	49106	M1-like macrophage	Human	R&D Systems	MAB392	AB_2261214	1
150Nd	CD86	IT2.2	M1-like macrophage	Human	Fluidigm	3150020B	AB_2661798	1
151Eu	CXCL10	Polyclonal	M1-like macrophage	Human	Novus Biologicals	AF-266-NA	AB_354433	1
145Nd	CD163	GHI/61	M2-like macrophage	Human	Fluidigm	3145010B	N/A	1
148Nd	Arginase 1/A RG1	658922	M2-like macrophage	Human	R&D Systems	MAB58681	N/A	1
162Dy	beta 2-Microglobulin (B2M)	883028	M2-like macrophage	Human	R&D Systems	MAB8248	N/A	1
168Er	CD206	15-2	M2-like macrophage	Human	Fluidigm	3168008B	AB_2661805	1
141Pr	CD326/ EpCAM	9C4	Epithelial marker	Human	Fluidigm	3141006B	AB_2687653	2
171Yb	KRT19/CK19	Polyclonal	Epithelial marker	Human/ Mouse	Novus Biologicals	NB100-687	AB_2265512	1
209Bi	Vimentin	2D1	Mesenchymal marker	Human/ Mouse	Novus Biologicals	NBP1-92687	AB_11017879	1
155Gd	Fibronectin	2F4	Mesenchymal marker	Human	Thermo Fisher Scientific	MA5-17075	AB_2538546	1
156Gd	CD90.2	30-H12	Stromal fibroblast	Mouse	Fluidigm	3156006B	AB_2801433	1
165Ho	CD31	390	Endothelial	Mouse	Fluidigm	3165013B	AB_2801434	1
176Yb	c-Myc	9E10	Oncogenic marker	Human	Fluidigm	3176012B	AB_2756296	1
172Yb	EGFP	Polyclonal	Labeling tag	N/A	R&D Systems	AF4240	AB_884445	2

*Antibody dilutions were prepared in total 50 μl antibody cocktail.

Supplementary Table 4. Clinicopathological information and percentages of hybrid categories of prostate cancer patients with primary tumors. Related to Fig. 5 and Supplementary Fig. 6

Sample ID	PSA level	Metastasis (MET)	Gleason score	TNM staging	Category I: no hybrid cell (%)	Category II: low hybrid cell (%)	Category III: high hybrid cell (%)
P01	111	Y	4+5	TxN1M1b, IVB	90	10	0
P02	30	N	3+4	T3bN0M0, IIIB	90	10	0
P03	16	Y	4+5	T3bN0M1b, IVB	90	10	0
P04	137	Y	4+5	T4N1M1b, IVB	50	50	0
P05	7	N	4+3	T2N0M0, IIB	90	10	0
P06	47	N	4+3	T3bN0M0, IIIB	40	50	10
P07	259	Y	4+5	T4N1M1b, IVB	20	70	10
P08	46	N	3+3	T2cN0M0, IIIA	90	10	0
P09	18	N	3+4	T3bN0M0, IIIB	60	40	0
P10	63	N	4+5	T3bN0M0, IIIC	50	50	0
P11	13	N	3+4	T3aN0M0, III	90	10	0
P12	10	N	3+5	T2N0M0, IIC	100	0	0
P13	83	Y	3+4	T3aN0M1b, IVB	70	30	0
P14	22	N	3+3	T2cN0M0, IIIA	90	10	0
P15	1950	Y	4+4	T3aN1M1b, IVB	50	50	0
P16	125	Y	4+3	T3aN0M1b, IVB	80	20	0
P17	18	Y	4+4	TxNxM1, IVB	60	40	0
P18	120	N	4+3	T3aN0M0, IIIB	60	40	0
P19	48	Y	3+4	T3aN0M1b, IVB	90	10	0
P20	16	N	5+4	T3bN0M0, IIIC	60	40	0
P21	57	Y	4+3	T3bN1M1b, IVB	80	20	0
P22	19	N	3+4	T3aN0M0, IIIB	90	10	0
P23	65	N	4+3	T3bN0M0, IIIB	70	30	0
P24	21	N	3+4	T3aN0M0, IIIB	100	0	0
P25	10	N	3+5	T2N0M0, IIC	90	10	0
P26	308	Y	4+5	T3bN1M1b, IVB	100	0	0
P27	21	N	3+4	T2N0M0, IIIA	80	20	0
P28	10	Y	4+4	T4 N0 M1b, IVB	60	40	0
P29	14	N	3+3	T3aN0M0, IIIB	80	20	0
P30	327	Y	4+3	T3bN1M1b, IVB	90	10	0
P31	37	N	3+4	T2cN0M0, IIIA	40	50	10
P32	5	N	5+4	TxNxM0, I	80	20	0
P33	73	N	4+4	T3bN,M0, IIIB	70	30	0
P34	322	Y	3+3	T4N1M1b, IVB	70	30	0
P35	20	N	3+3	T2cN0M0, IIIA	70	30	0
P36	9	N	5+5	T3bN1M0, IVA	30	40	30
P37	>1000	Y	4+4	T4N1M1c, IVB	0	80	20
P38	65	Y	4+3	T3bN1M1b, IVB	50	50	0
P39	9	N	4+5	T3bN1M0, IVA	80	20	0
P40	95	N	4+4	T2N0M0, IIIA	80	20	0
P41	977	Y	4+3	T3bN1M1b, IVB	60	40	0
P42	146	N	3+4	T3bN0M0, IIIB	50	50	0
P43	1	Y	4+5	cT4N1M1b, IVB	70	30	0
P44	24	N	3+5	T2N0M0, IIIA	90	10	0
P45	12	N	3+3	T2cN0M0, IIA	70	30	0
P46	66	N	5+4	T3bN0M0, IIIC	70	30	0
P47	213	Y	5+5	TxNxM1b, IVB	100	0	0

P48	28	N	3+3	T3aN0M0, IIIB	80	20	0
P49	8	N	3+3	T2cN0M0, IIA	100	0	0
P50	>1000	Y	4+5	TxNxM1b, IVB	90	10	0

Note: Samples from the same patient: P12 and P25.

Supplementary Table 5. Clinicopathological information and percentages of hybrid categories of prostate cancer patients with metastatic tumors. Related to Fig. 5 and Supplementary Fig. 6

Sample ID	PSA level	Metastasis (MET)	Site of metastasis	Gleason score	TNM staging	Category I: no hybrid cell (%)	Category II: low hybrid cell (%)	Category III: high hybrid cell (%)
M01	84	Y	LN* (regional)	4 + 5	T3aN1M0, IVA	20	80	0
M02	6	Y	LN* (regional)	4 + 4	T3aN1M0, IVA	10	90	0
M04	15	Y	LN* (regional)	4 + 3	T3aN1M0, IVA	20	70	10
M05	62	Y	LN* (regional)	4 + 4	T3aN1M1, IVB	0	70	30
M06	371	Y	Bone	4 + 4	T4N1M1, IVB	10	80	10
M07	11	Y	LN* (regional)	5 + 5	T3bN1M0, IVA	40	60	0
M09	89	Y	LN* (distal)	4 + 4	TxN1M1a, IVB	40	50	10
M10	9	Y	Bone	5 + 5	T4N0M1b, IVB	10	60	30
M11	> 1000	Y	Bone	4 + 3	TxN1M1c, IVB	0	10	90
M12	25	Y	Bone	4 + 4	TxN1M1b, IVB	40	60	0
M13	95	Y	Bone	4 + 4	T4N1M1, IVB	0	50	50
M14	48	Y	Bone	4 + 4	T4N1M1, IVB	20	70	10
M15	0.21	Y	LN* (pelvic)	3 + 4	T2N1M0, IVA	50	50	0
M16	111	Y	LN*	3 + 4	TxN1M1b, IVB	20	80	0
M17	63	Y	LN* (left neck)	4 + 4	TxN1M1b, IVB	70	30	0
M18	145	Y	LN* (mediastinal)	4 + 4	T3bN1M1b, IVB	0	90	10
M19	339	Y	LN*	4 + 4	TxN1M1c, IVB	60	40	0
M20	1300	Y	Lung	4 + 3	T3N0M1c,IVB	70	30	0
M21	13600	Y	Brain	4 + 5	TxN1M1b, IVB	20	80	0
M22	6490	Y	LN* (left inguinal)	4 + 4	T2N1M0, IVA	60	40	0
M23	> 1000	Y	Bone	5 + 3	T3bN0M1b, IVB	20	80	0
M25	288	Y	Bone	4 + 4	T3a-bNxM1b, IVB	30	70	0
M26	601	Y	Bone	5 + 4	T4N1M1b, IVB	10	40	50
M27	1920	Y	LN* (mediastinal)	4 + 5	TxN1M1c,IVB	30	60	10
M28	830	Y	Bone	4 + 4	T2NxM1b, IVB	0	80	20
M29	8030	Y	Bone	3 + 4	TxNxM1b,IVB	20	60	20
M30	287	Y	Bone	5 + 4	T4N1M1b, IVB	60	30	10
M31	7	Y	LN* (right neck)	5 + 5	T4 N1 M1b, IVB	10	50	40
M32	364	Y	Lung	4 + 4	T3bN0M1c, IVB	20	70	10
M33	23	Y	Bone	4 + 4	T3aN0M1b, IVB	50	30	20
M34	0.84	Y	LN* (left pelvic)	4 + 3	T3bN1M0, IVA	70	30	0
M35	357	Y	LN* (IMA)	4 + 5	T3bN1M1c, IVB	0	60	40
M37	517	Y	LN* (left neck)	5 + 4	TxN1M1c, IVB	10	80	10
M38	42	Y	LN* (right pelvic)	4 + 4	T3bN1M0, IVA	80	20	0
M39	42	Y	LN* (left pelvic)	4 + 4	T3bN1M0, IVA	40	60	0
M40	237	Y	Urinary bladder	4 + 5	T4N0M1b, IVB	0	80	20
M41	7	Y	Bone	3 + 4	T3N0M1b, IVB	40	60	0
M43	155	Y	Bone	4 + 4	TxNxM1, IVA	30	70	0
M44	633	Y	Bone	5 + 4	TXNXM1b, IVB	40	50	10
M45	645	Y	Bone	4 + 4	TxNxM1b, IVB	70	30	0
M46	79	Y	Colon	5 + 5	T3bN0M1c, IVB	0	20	80
M47	3850	Y	Liver	4 + 4	T3bN0M1c, IVB	30	40	30
M49	3153	Y	Bone	4 + 3	TxN1M1b, IVB	30	70	0
M51	1	Y	LN* (inguinal)	4 + 5	T2N1M0, IVA	20	80	0

LN*: Lymph node
Note: Samples from the same patient: M13 and M14, M21 and M49, M26 and M30, and M38 and M39.

Supplementary Table 6. CyTOF antibody panel for human peripheral blood mononuclear cells (PBMCs) and IBIDI circulation test. Related to Fig. 8 and Supplementary Fig. 8

Tag	Antigen	Clone	Marker type	Reactivity	Vendor	Cat. No.	RRID	Amount (µl)*
89Y	CD45	HI30	Leukocyte	Human	Fluidigm	3089003B	AB_2661851	1
141Pr	CD3	UCHT1	Leukocyte /T cell	Human	Fluidigm	3141019B	N/A	1
142Nd	CD19	HIB19	Leukocyte /B cell	Human	Fluidigm	3142001B	AB_2651155	1
148Nd	CD16	3G8	Leukocyte /monocyte/macrophage	Human	Fluidigm	3148004B	AB_2661791	1
175Lu	CD14	M5E2	Leukocyte /monocyte/macrophage	Human	Fluidigm	3175015B	AB_2811083	1
176Yb	CD56	CMSSB	Leukocyte /NK cell	Human	Fluidigm	3176003B	AB_2756430	1
162Dy	CD66b	80H3	Leukocyte/neutrophils	Human	Fluidigm	3162023B	N/A	1
150Nd	CD86	IT2.2	M1-like macrophage	Human	Fluidigm	3150020B	AB_2661798	1
145Nd	CD163	GHI/61	M2-like macrophage	Human	Fluidigm	3145010B	N/A	1
171Yb	Keratin 19	Polyclonal	Epithelial marker	Human/Mouse	Novus	NB100-687	AB_2265512	1
173Yb	EpCAM	Polyclonal	Epithelial marker	Human	R&D systems	AF960	AB_355745	2
112Cd	MUC-1	604804	Epithelial marker	Human	R&D Systems	MAB6298	AB_10640403	1
156Gd	Vimentin	RV202	Mesenchymal marker	Human	Fluidigm	3156023A	N/A	1
155Gd	Fibronectin	2F4	Mesenchymal marker	Human	Thermo Fisher Scientific	MA5-17075	AB_2538546	1
164Dy	Kallikrein 3/ PSA	SPM352	Prostate marker	Human	Novus Biologicals	NBP2-34768	N/A	2
113Cd	PSMA	460420	Prostate marker	Human	R&D Systems	MAB4234	AB_10889642	2

*Antibody dilutions were prepared in total 50 µl antibody cocktail.

Supplementary Table 7. Clinicopathological information of prostate cancer patients in human PBMC CyTOF study. Related to Fig. 8

Patient ID	PSA level	Biochemical recurrent (BCR)	Castration resistant (CR)	Metastasis (MET)	Site of metastasis	Gleason score	TNM staging
BCR only group							
115	0.20	Y	N	N	N/A	4+4	T3b, IIIB
133	0.78	Y	N	N	N/A	4+4	T3bN0M0R1, IIIB
120	0.96	Y	N	N	N/A	4+5	T3aN1M0, IVA
124	1.32	Y	N/A	N	N/A	3+4	T2c, IIB
117	5.84	Y	N	N	N/A	4+4	T1cNxMx, IIC
118	9.64	Y	N	N	N/A	3+3	TxNxMx, I
130	9.66	Y	N	N	N/A	3+4	T2bNxMx, IIB
CR/MET group							
092	0.26	Y	Y	N	N/A	4+4	T3bN0Mx, IIIB
126	0.37	Y	Y	N	N/A	4+3	T3N0Mx, IIIB
139	0.73	Y	Y	N	N/A	3+4	T1cNxMx, IIB
114	7.22	Y	Y	N	N/A	3+4	T1c, IIB
122	0.05	N/A	N	Y	Lymph node	4+4	T3bN1M0R1, IVA
128	43.84	N/A	N	Y	Lymph node	4+5	T1cN1M0, IVA
054	1.39	N/A	Y	Y	Bone, lung	4+5	T4N0M1, IVB
123	8.64	Y	Y	Y	Bone	4+3	T2cN0M1R1, IVB
083	10.06	N/A	Y	Y	Bone	4+4	T2bN1/2M1, IVB

References

1. Song H, *et al.* Single-cell analysis of human primary prostate cancer reveals the heterogeneity of tumor-associated epithelial cell states. *Nat Commun* **13**, 1-20 (2022).

2. Feng E, *et al.* Intrinsic molecular subtypes of metastatic castration-resistant prostate cancer. *Clin Cancer Res* **28**, 5396-5404 (2022).

3. Biswas SK, Mantovani A. Macrophage plasticity and interaction with lymphocyte subsets: cancer as a paradigm. *Nat Immunol* **11**, 889-896 (2010).

4. Locati M, Curtale G, Mantovani A. Diversity, mechanisms, and significance of macrophage plasticity. *Annu Rev Pathol* **15**, 123-147 (2020).

5. Mantovani A, Locati M. Tumor-associated macrophages as a paradigm of macrophage plasticity, diversity, and polarization: lessons and open questions. *Arterioscler Thromb Vasc Biol* **33**, 1478-1483 (2013).

6. Sica A, Mantovani A. Macrophage plasticity and polarization: in vivo veritas. *J Clin Invest* **122**, 787-795 (2012).

7. Jablonski KA, *et al.* Novel markers to delineate murine M1 and M2 macrophages. *PLoS One* **10**, e0145342 (2015).

8. Orecchioni M, Ghosheh Y, Pramod AB, Ley K. Macrophage polarization: different gene signatures in M1 (LPS+) vs. classically and M2 (LPS–) vs. alternatively activated macrophages. *Front Immunol* **10**, 1084 (2019).

9. Jayasingam SD, Citartan M, Thang TH, Mat Zin AA, Ang KC, Ch'ng ES. Evaluating the polarization of tumor-associated macrophages into M1 and M2 phenotypes in human cancer tissue: technicalities and challenges in routine clinical practice. *Front Oncol* **9**, 1512 (2019).

10. Miki S, Suzuki JI, Takashima M, Ishida M, Kokubo H, Yoshizumi M. S-1-Propenylcysteine promotes IL-10-induced M2c macrophage polarization through prolonged activation of IL-10R/STAT3 signaling. *Sci Rep* **11**, 22469 (2021).

11. Yu T, *et al.* Modulation of M2 macrophage polarization by the crosstalk between Stat6 and Trim24. *Nat Commun* **10**, 1-15 (2019).

12. Abel AM, Yang C, Thakar MS, Malarkannan S. Natural killer cells: development, maturation, and clinical utilization. *Front Immunol* **9**, 1869 (2018).

13. Tu TC, *et al.* CD160 is essential for NK-mediated IFN-gamma production. *J Exp Med* **212**, 415-429 (2015).

14. Lee LJ, *et al.* Differential regulation of NK cell receptors in acute lymphoblastic leukemia. *J Immunol Res* **2022**, 7972039 (2022).

15. Yang C, *et al.* Heterogeneity of human bone marrow and blood natural killer cells defined by single-cell transcriptome. *Nat Commun* **10**, 3931 (2019).

16. Guo H, Cruz-Munoz ME, Wu N, Robbins M, Veillette A. Immune cell inhibition by SLAMF7 is mediated by a mechanism requiring src kinases, CD45, and SHIP-1 that is defective in multiple myeloma cells. *Mol Cell Biol* **35**, 41-51 (2015).

17. Hegde S, Leader AM, Merad M. MDSC: Markers, development, states, and unaddressed complexity. *Immunity* **54**, 875-884 (2021).

18. Talmadge JE, Gabrilovich DI. History of myeloid-derived suppressor cells. *Nat Rev Cancer* **13**, 739-752 (2013).

19. Damuzzo V*, et al.* Complexity and challenges in defining myeloid-derived suppressor cells. *Cytometry B Clin Cytom* **88**, 77-91 (2015).

20. Zhao Y, Wu T, Shao S, Shi B, Zhao Y. Phenotype, development, and biological function of myeloid-derived suppressor cells. *Oncoimmunology* **5**, e1004983 (2016).

21. Veglia F, Sanseviero E, Gabrilovich DI. Myeloid-derived suppressor cells in the era of increasing myeloid cell diversity. *Nat Rev Immunol* **21**, 485-498 (2021).

22. Subramanian A*, et al.* Gene set enrichment analysis: a knowledge-based approach for interpreting genome-wide expression profiles. *Proc Natl Acad Sci USA* **102**, 15545-15550 (2005).

23. Liberzon A, Subramanian A, Pinchback R, Thorvaldsdottir H, Tamayo P, Mesirov JP. Molecular signatures database (MSigDB) 3.0. *Bioinformatics* **27**, 1739-1740 (2011).

24. Liberzon A, Birger C, Thorvaldsdottir H, Ghandi M, Mesirov JP, Tamayo P. The Molecular Signatures Database (MSigDB) hallmark gene set collection. *Cell Syst* **1**, 417-425 (2015).
