# Supplementary material for: Phagocytosis-initiated tumor hybrid cells acquire a c-Myc-mediated quasi-polarization state for immunoevasion and distant dissemination
Source: Nat Commun. 2023 Oct 17;14:6569. doi: 10.1038/s41467-023-42303-5 (PMC10582093; doi:10.1038/s41467-023-42303-5)
Supplement: Supplementary file 3 — Reporting Summary [file 41467_2023_42303_MOESM3_ESM.pdf]

nature portfolio

Corresponding author(s): Tim Hui-Ming Huang
Zhao Zhang
Chun-Liang Chen

Last updated by author(s): 9/26/2023

Reporting Summary

Nature Portfolio wishes to improve the reproducibility of the work that we publish. This form provides structure for consistency and transparency in reporting. For further information on Nature Portfolio policies, see our Editorial Policies and the Editorial Policy Checklist.

Statistics

For all statistical analyses, confirm that the following items are present in the figure legend, table legend, main text, or Methods section.

n/a	Confirmed	
☐	☒	The exact sample size (*n*) for each experimental group/condition, given as a discrete number and unit of measurement
☒	☐	A statement on whether measurements were taken from distinct samples or whether the same sample was measured repeatedly
☐	☒	The statistical test(s) used AND whether they are one- or two-sided<br>*Only common tests should be described solely by name; describe more complex techniques in the Methods section.*
☒	☐	A description of all covariates tested
☒	☐	A description of any assumptions or corrections, such as tests of normality and adjustment for multiple comparisons
☐	☒	A full description of the statistical parameters including central tendency (e.g. means) or other basic estimates (e.g. regression coefficient) AND variation (e.g. standard deviation) or associated estimates of uncertainty (e.g. confidence intervals)
☐	☒	For null hypothesis testing, the test statistic (e.g. *F*, *t*, *r*) with confidence intervals, effect sizes, degrees of freedom and *P* value noted<br>*Give P values as exact values whenever suitable.*
☒	☐	For Bayesian analysis, information on the choice of priors and Markov chain Monte Carlo settings
☒	☐	For hierarchical and complex designs, identification of the appropriate level for tests and full reporting of outcomes
☐	☒	Estimates of effect sizes (e.g. Cohen's *d*, Pearson's *r*), indicating how they were calculated

Our web collection on statistics for biologists contains articles on many of the points above.

Software and code

Policy information about availability of computer code

Data collection	Illumina output was converted to fastq format using Bcl2fastq v2.17.
Data analysis	Softwares: R (v4.2.2), R studio (v2022.07.2+576), Prism (v9.4.0), CyTOF (v7.0), Fiji ImageJ and FlowJo (v10.8.1)<br>cytofkit2 and PhenoGraph were used to analyze CyTOF data.<br>10X Genomics Space Ranger tool, kb-python (v0.26.4), AUCell (v1.20.1), doMC (v1.3.8), ggplot2 (v3.4.0), RVAv2 (v2.0.0), Seurat (v4.3.0), and SeuratObject (v4.1.3) were used to analyze spatial transcriptomics data.

For manuscripts utilizing custom algorithms or software that are central to the research but not yet described in published literature, software must be made available to editors and reviewers. We strongly encourage code deposition in a community repository (e.g. GitHub). See the Nature Portfolio guidelines for submitting code & software for further information.

Data

Policy information about availability of data

All manuscripts must include a data availability statement. This statement should provide the following information, where applicable:

- Accession codes, unique identifiers, or web links for publicly available datasets
- A description of any restrictions on data availability
- For clinical datasets or third party data, please ensure that the statement adheres to our policy

The GEO accession number for spatial transcriptomics reported in this paper is GSE194102. To review GEO accession: https://www.ncbi.nlm.nih.gov/geo/query/

acc.cgi?acc=GSE194102. The digital gene-cell matrices of GSE176031 were downloaded from GEO website (https://www.ncbi.nlm.nih.gov/geo/query/acc.cgi?acc=GSE176031). The Microarray datasets Grasso, Taylor, Varambally were retrieved from GSE35988 (https://www.ncbi.nlm.nih.gov/geo/query/acc.cgi?acc=GSE35988), GSE21034 (https://www.ncbi.nlm.nih.gov/geo/query/acc.cgi?acc=GSE21034), and GSE3325 (https://www.ncbi.nlm.nih.gov/geo/query/acc.cgi?acc=GSE3325), respectively. The TCGA PRAD dataset was retrieved from cBioPortal (https://www.cbioportal.org/). All other data supporting the findings of this study are available from the corresponding author upon reasonable request.

Human research participants

Policy information about studies involving human research participants and Sex and Gender in Research.

Reporting on sex and gender	All the research participants are men because prostate cancer only occurs in males.
Population characteristics	Two cohorts of participants with prostate cancer were enrolled for IHC and PBMC studies: The cohort for IHC study includes patients with primary and/or metastatic prostate tumors. And the cohort for PBMC study was specific to patients with biochemical recurrence. The related clinicopathological information of individual patients is reported in Supplementary Table 4, 5, and 7.
Recruitment	For the cohort for IHC study, the pathologists Drs. C-C Chen and C-W Hsu (co-authors) collected samples from patients eligible for IHC study. For the cohort for PBMC study, the urologist Dr. Liss (co-author) and his research coordinator screened patients for study eligibility. Prostate cancer patients with biochemical recurrence were recruited before their initial androgen deprivation therapy. Informed consents of both cohorts were obtained from all patients.
Ethics oversight	The Institutional Review Board at the University of Texas Health Science Center at San Antonio and Ditmanson Medical Foundation Chia-Yi Christian Hospital.

Note that full information on the approval of the study protocol must also be provided in the manuscript.

Field-specific reporting

Please select the one below that is the best fit for your research. If you are not sure, read the appropriate sections before making your selection.

[x] Life sciences [ ] Behavioural & social sciences [ ] Ecological, evolutionary & environmental sciences

For a reference copy of the document with all sections, see nature.com/documents/nr-reporting-summary-flat.pdf

Life sciences study design

All studies must disclose on these points even when the disclosure is negative.

Sample size	No sample size calculation was performed. Sample size was determined from similar experiments in the literature. Experiments were performed with a minimum of three biological replicates when possible.
Data exclusions	In single-cell RNA-seq analysis, for the quality control purposes, cells with low quality (less than 300 genes, 500 transcripts, or a mitochondrial level of 20% or greater) were filtered out as described in Methods section.
Replication	Experiments were carried out in three independent biological replicates when possible and were reproducible.
Randomization	Human specimen were allocated into groups according to disease status (biochemical recurrence only, castration resistance or metastasis). For functional studies, cells were subjected to different treatment and were allocated to different groups accordingly. Mice were allocated randomly for xenograft experiment groups.
Blinding	Blinding was not relevant to our study.

Reporting for specific materials, systems and methods

We require information from authors about some types of materials, experimental systems and methods used in many studies. Here, indicate whether each material, system or method listed is relevant to your study. If you are not sure if a list item applies to your research, read the appropriate section before selecting a response.

Materials & experimental systems

n/a	Involved in the study
	☒ Antibodies
	☒ Eukaryotic cell lines
☒	Palaeontology and archaeology
	☒ Animals and other organisms
☒	Clinical data
☒	Dual use research of concern

Methods

n/a	Involved in the study
☒	ChIP-seq
	☒ Flow cytometry
☒	MRI-based neuroimaging

Antibodies

Antibodies used

Mouse monoclonal anti-human CD47 (clone B6H12, eBioscience, Cat#14-0479-82)
Mouse monoclonal anti-human CD24 (clone SN3, Novus Biologicals, Cat#NB100-64861)
Mouse monoclonal anti-human PD-L1 (clone 29E.2A3, BioXCell, Cat#BE0285)
Mouse monoclonal anti-human BCL-2 Alexa Fluor 647 (clone 100, Biolegend, Cat#658706)
Mouse monoclonal anti-human CD86 PE (clone IT2.2, Biolegend, Cat#305406)
Mouse monoclonal anti-human CD206 Brilliant Violet 605 (clone 15-2, Biolegend, Cat#321140)
Mouse monoclonal anti-human CD4-PE (clone RPA-T4, Biolegend, Cat# 300508)
Mouse monoclonal anti-human/Cynomolgus/Rhesus CD8-Alexa Fluor 647 (clone SK1, Biolegend, Cat# 344726)
Mouse monoclonal anti-human CD25-FITC (clone BC96, Biolegend, Cat# 302604)
Mouse monoclonal anti-human CD45-Brilliant Violet 785 (clone HI30, Biolegend, Cat#305406 304047)
Rat anti-mouse F4/80-PE (clone: A3-1, Thermo Fisher Scientific, Cat#MA5-16631)
Mouse monoclonal anti-human c-Myc-Alexa Fluor 647 (clone 9E10, Biolegend, Cat#626810)
Rabbit recombinant multiclonal anti-human CD47 (clone: RM1014, Abcam, ab284132)
Rabbit monoclonal anti-human GAPDH (clone 14C10, Cell Signaling Technology, Cat#2118S)
Mouse anti-rabbit IgG-HRP (Santa Cruz Biotechnology, Cat#sc-2357)
Goat anti-mouse IgG-HRP (Santa Cruz Biotechnology, Cat#sc-2005)
Mouse monoclonal anti-human BCL2 (clone 118701, R&D Systems, Cat#MAB827)
Rabbit polyclonal anti-human/mouse/rat phospho-LaminA/C (Ser22, Cell Signaling Technology, Cat#2026S)
Mouse monoclonal anti-human/mouse/rat β-Actin (clone 937215, R&D Systems, Cat#MAB8929).
Rabbit monoclonal anti-human/mouse/rat SIRPα/SHPS1 (clone D6I3M, Cell Signaling Technology, Cat#13379)
Rabbit monoclonal anti-human CD14 (clone SP192, Abcam, Cat#ab183322)
Rabbit polyclonal anti-human/mouse/rat EpCAM (Abcam, Cat#ab71916)
Mouse monoclonal anti-human/mouse/rat Vimentin (clone 2D1, Novus Biological, Cat#NBP192687)
Rabbit monoclonal anti-human/monkey CD68 (clone: D4B9C, Cell Signaling Technology, Cat#CST76437)
Rabbit monoclonal anti-human/monkey CD86 (clone:E2G8P, Cell Signaling Technology, Cat#CST91882)
Mouse monoclonal anti-human EpCAM (clone: VU1D9, Cell Signaling Technology, Cat#CST2929)
Mouse monoclonal anti-human CD326 (EpCAM)-FITC (clone VU-1D9, STEMCELL Technologies, Cat# 60136Fl)
Mouse monoclonal anti-human Cytokeratin 18-Alexa Fluor 647 (clone: DA-7, Biolegend, Cat#628404)
Mouse monoclonal anti-human/mouse/monkey pan-Cytokeratin (clone:C11, Cell Signaling Technology, Cat#CST4545)
Rat monoclonal anti-mouse anti-F4/80 (clone D2S9R, Cell Signaling Technology, Cat#70076S)
Mouse monoclonal anti-human pan-cytokeratin cocktail (clone: AE1/AE3, Sakura Finetek USA, Cat#60-0022)
Mouse monoclonal anti-human CD68 (clone: KP1, Zeta Corporation, Cat#50-221-5864)
Rabbit monoclonal anti-mouse F4/80 (clone SP115, ThermoFisher Scientific, Cat#MA5-16363)
Goat anti-mouse IgG H&L Alexa Fluor 647 (Abcam, Cat#ab150115)
Goat anti-mouse IgG H&L Alexa Fluor 594 (Abcam, Cat#ab150116)
Goat anti-rat IgG H&L Alexa Fluor 594 (Abcam, Cat#ab150168)
Goat anti-rabbit IgG H&L Alexa Fluor 488 (Abcam, Cat#ab150077)
Goat anti-rabbit IgG H&L Alexa Fluor 647 (Abcam, Cat#ab150087)
Mouse monoclonal anti-human c-Myc (clone 9E10, R&D Systems, Cat#MAB3696)
Rabbit polyclonal anti-human c-Myc (ThermoFisher Scientific, Cat#PA5-85185)
Antibodies for CyTOF analysis, see Supplementary Table 3 and 6.

Validation

Antibodies were validated by the manufacturers as indicated in the data sheets, cited in multiple peer reviewed publications, and further validated in our experiments.

Eukaryotic cell lines

Policy information about cell lines and Sex and Gender in Research

Cell line source(s)

22Rv1 (ATCC, Cat#CRL-2505), C4-2 (ATCC, Cat#CRL-3314), DU145 (ATCC, Cat#HTB-81), TRAMP-C2 (ATCC, Cat#CRL-2731), RM-1 (ATCC, Cat#CRL-3310), human peripheral blood mononuclear cells (PBMCs) (ATCC, Cat# PCS-800-081), Peripheral blood CD14+ monocyte cells (STEMCELL Technologies, Cat#70035), U937 (ATCC, Cat# CRL-1593.2), and IC-21 (ATCC, Cat#TIB-186)

Authentication

The authentication was done by the manufacture (22Rv1, C4-2, DU145, TRAPM-C2, RM-1, U937, IC-21, and human PBMCs

	were authenticated by ATCC and Peripheral blood CD14+ monocyte cells were authenticated by STEMCELL Technologies).
Mycoplasma contamination	Cell lines were not tested for mycoplasma contamination. No indication of contamination was observed.
Commonly misidentified lines (See ICLAC register)	Only U937 is listed in Table 2 of ICLAC and might have cross-contamination. However, we obtained this cell line from the ATCC-authenticated stock CRL-1593.2.

Animals and other research organisms

Policy information about studies involving animals; ARRIVE guidelines recommended for reporting animal research, and Sex and Gender in Research

Laboratory animals	Male Nu/Nu mice (The Jackson Laboratory, JAX: 007850) and male C57BL/6J (The Jackson Laboratory, JAX: 000664) mice
Wild animals	No wild animals were used in this study.
Reporting on sex	Only male mice were used due to prostate cancer occurrence in males.
Field-collected samples	No field-collected samples were used in this study.
Ethics oversight	Institutional Animal Care and Use Committee at the University of Texas Health Science Center at San Antonio, protocol number ORCA20200124AR.

Note that full information on the approval of the study protocol must also be provided in the manuscript.

Flow Cytometry

Plots

Confirm that:

- [x] The axis labels state the marker and fluorochrome used (e.g. CD4-FITC).
- [x] The axis scales are clearly visible. Include numbers along axes only for bottom left plot of group (a 'group' is an analysis of identical markers).
- [x] All plots are contour plots with outliers or pseudocolor plots.
- [x] A numerical value for number of cells or percentage (with statistics) is provided.

Methodology

Sample preparation	For phagocytosis assay, co-cultured single-cell suspensions were stained with Zombie-NIR dye (Biolegend, 423106) for 20 minutes to exclude the dead cells. After washing with Cell Staining Buffer (Biolegend, 420201), cells were pre-incubated with Human TruStain FcX (Biolegend, 422302) for 10 minutes to block non-specific staining, and then incubated with fluorescence-labeled antibodies for 30 minutes at 4°C in darkness.<br>For CyTOF, single-cell suspensions from co-cultured cells, tumors, or PBMCs were incubated with Cell-ID Cisplatin (Fluidigm, 201064) at room temperature for 5 minutes. Cells were then fixed with 3.2% paraformaldehyde (Thermo Fisher Scientific, 28906) and washed with Maxpar Cell Staining Buffer (Fluidigm, 201068). Cells were then incubated with Maxpar Perm-S buffer (Fluidigm, 201066) and TruStain Fc-X (Biolegend, 422302). The cell surface and cytosol antibodies were pooled and added to the cells for incubation at room temperature for 1 hour. After washing with Maxpar Cell Staining Buffer (Fluidigm, 201068), cell pellets were chilled on ice for 10 minutes, gently resuspended in cold methanol and incubated on ice for another 10 minutes. After washing, cells were stained with the pooled nuclear antibodies at room temperature for 30 minutes, and then incubated with Maxpar Fix and Perm Buffer (Fluidigm, 201067) containing 0.125 mM Cell-ID Intercalator-Ir 191/193 (Fluidigm, 201192A) at 4°C for 16-18 hours.
Instrument	BD FACSCelesta Cell Analyzer (BD Biosciences), BD FACSAria Fusion Cell Sorter (BD Biosciences), and Helios third-generation mass cytometer (Fluidigm).
Software	Prism (v10.0.0(131)), FlowJo (v10.8.1), and CyTOF software (v7.0, Fluidigm).
Cell population abundance	Putative human hybrid tumor cells were sorted by mCherry+/EGFP+ through BD FACSAria Fusion Cell Sorter (BD Biosciences), the population size was ~1%.
Gating strategy	For gating the hybrid cells, FSC_Zombie negative cells were firstly gated as live cells. PE_CD86+/FITC_EGFP- or PE_F4/80+/PB_Tag-it-Violet- population was identified as macrophages or monocytes, PE_CD86-/FITC_EGFP+ or PE_F4/80-/PB_Tag-it-Violet+ population was identified as cancer cells, and PE_CD86+/FITC_EGFP+ or PE_F4/80+/PB_Tag-it-Violet+ population was identified as putative hybrid cells. For detail gating strategies, see Figures 1d, Supplementary Figure 1d, Supplementary Figure 4f, Supplementary Figure 5b, Supplementary Figure 5e, and Supplementary Figure 10.

- [x] Tick this box to confirm that a figure exemplifying the gating strategy is provided in the Supplementary Information.
